# Genetic Downregulation of the Metabotropic Glutamate Receptor Type 5 Dampens the Reactive and Neurotoxic Phenotype of Adult ALS Astrocytes

**DOI:** 10.3390/cells12151952

**Published:** 2023-07-27

**Authors:** Carola Torazza, Francesca Provenzano, Elena Gallia, Maria Cerminara, Matilde Balbi, Tiziana Bonifacino, Sara Tessitore, Silvia Ravera, Cesare Usai, Ilaria Musante, Aldamaria Puliti, Ludo Van Den Bosch, Paymaan Jafar-nejad, Frank Rigo, Marco Milanese, Giambattista Bonanno

**Affiliations:** 1 Department of Pharmacy (DIFAR), University of Genoa, Viale Cembrano 4, 16148 Genova, Italy; carola.torazza@unige.it (C.T.); francescaprovenzano.fp@gmail.com (F.P.); elena.gallia@libero.it (E.G.); matilde.balbi@unige.it (M.B.); tiziana.bonifacino@unige.it (T.B.); sara.tessitore@edu.unige.it (S.T.); giambattista.bonanno@unige.it (G.B.); 2 Department of Neurosciences, Rehabilitation, Ophthalmology, Genetics, Maternal and Child Health (DINOGMI), University of Genoa, Largo Paolo Daneo, 16132 Genoa, Italy; maria.cerminara@edu.unige.it (M.C.); aldamaria.puliti@unige.it (A.P.); 3 UOC Genetica Medica, IRCCS Istituto Giannina Gaslini, 16147 Genoa, Italy; ilaria.musante@unige.it; 4 Inter-University Center for the Promotion of the 3Rs Principles in Teaching & Research (Centro 3R), 56122 Pisa, Italy; 5 Department of Experimental Medicine (DIMES), University of Genoa, Via Alberti L.B. 2, 16132 Genova, Italy; silvia.ravera@unige.it; 6 Institute of Biophysics, National Research Council (CNR), Via De Marini 6, 16149 Genoa, Italy; cesare.usai@ibf.cnr.it; 7 Department of Neurosciences, Experimental Neurology, and Leuven Brain Institute, KU Leuven-University of Leuven, 3000 Leuven, Belgium; ludo.vandenbosch@kuleuven.be; 8 VIB-Center for Brain & Disease Research, Laboratory of Neurobiology, 3000 Leuven, Belgium; 9 Ionis Pharmaceuticals, Carlsbad, CA 92010, USA; pjafarne@ionisph.com (P.J.-n.); frigo@ionisph.com (F.R.); 10 IRCCS Ospedale Policlinico San Martino, Largo Rosanna Benzi 10, 16132 Genoa, Italy

**Keywords:** amyotrophic lateral sclerosis, mGlu5 receptor, adult mouse spinal cord astrocytes, *Grm5* genetic ablation, SOD1^G93A^ mice

## Abstract

Amyotrophic lateral sclerosis (ALS) is a fatal neurodegenerative disease characterized by progressive degeneration of motor neurons (MNs). Astrocytes display a toxic phenotype in ALS, which results in MN damage. Glutamate (Glu)-mediated excitotoxicity and group I metabotropic glutamate receptors (mGluRs) play a pathological role in the disease progression. We previously demonstrated that in vivo genetic ablation or pharmacological modulation of mGluR5 reduced astrocyte activation and MN death, prolonged survival and ameliorated the clinical progression in the SOD1^G93A^ mouse model of ALS. This study aimed to investigate in vitro the effects of mGluR5 downregulation on the reactive spinal cord astrocytes cultured from adult late symptomatic SOD1^G93A^ mice. We observed that mGluR5 downregulation in SOD1^G93A^ astrocytes diminished the cytosolic Ca^2+^ overload under resting conditions and after mGluR5 simulation and reduced the expression of the reactive glial markers GFAP, S100β and vimentin. In vitro exposure to an anti-mGluR5 antisense oligonucleotide or to the negative allosteric modulator CTEP also ameliorated the altered reactive astrocyte phenotype. Downregulating mGluR5 in SOD1^G93A^ mice reduced the synthesis and release of the pro-inflammatory cytokines IL-1β, IL-6 and TNF-α and ameliorated the cellular bioenergetic profile by improving the diminished oxygen consumption and ATP synthesis and by lowering the excessive lactate dehydrogenase activity. Most relevantly, mGluR5 downregulation hampered the neurotoxicity of SOD1^G93A^ astrocytes co-cultured with spinal cord MNs. We conclude that selective reduction in mGluR5 expression in SOD1^G93A^ astrocytes positively modulates the astrocyte reactive phenotype and neurotoxicity towards MNs, further supporting mGluR5 as a promising therapeutic target in ALS.

## 1. Introduction

Amyotrophic lateral sclerosis (ALS) is the most common adult-onset motor neuron (MN) disease characterized by the progressive degeneration of lower MNs in the brain stem and spinal cord, and upper corticospinal MNs in the motor cortex [1,2]. MN death leads to muscle wasting, weakness and spasticity, and death occurs between 2 and 5 years after diagnosis due to respiratory failure [3]. Several ALS patients also develop cognitive and behavioral impairments; more than 10% also present frontotemporal dementia [4,5].

ALS can occur in two forms, sporadic (sALS), representing more than 90% of cases, and familial (fALS), which is genetically transmissible and accounts for about 10% of cases. sALS and fALS exhibit similar pathological traits, possibly indicating the involvement of common mechanisms [6]. More than 30 mutated genes have been linked to ALS [7], 4 being responsible for more than 70% of all fALS cases. These are mutations in superoxide dismutase 1 (*SOD1*), TAR DNA-binding protein 43 (*TARDBP*) and fused in sarcoma protein (*FUS*), and hexanucleotide repeats in chromosome 9 open reading frame 72 (*C9ORF72*) [8]. The generation of transgenic animal models based on these genetic causes has represented a valuable tool leading to significant breakthroughs, and they also allowed preclinical studies in ALS [9].

Despite the dramatic progress of scientific knowledge, no effective drugs are available for the treatment of ALS. Riluzole, used since 1996 [10], and the Food and Drug Administration (FDA)-approved edaravone [11] produce only limited benefits in patients. Very recently, the FDA approved the antisense oligonucleotide Tofersen which reduces SOD1 synthesis in ALS patients with *SOD1* mutations [12,13].

Multiple pathological mechanisms have been associated with ALS. These include altered calcium (Ca^2+^) homeostasis, protein misfolding and aggregation, impaired axonal transport, mitochondrial dysfunction, oxidative stress, neuroinflammation, dysregulated RNA signaling and glutamate (Glu)-mediated excitotoxicity [14,15,16,17,18]. Moreover, the role of non-neuronal cells in the pathogenesis of ALS, particularly microglia, astrocytes, and oligodendrocytes, is broadly accepted [19,20], making ALS a non-cell-autonomous disease [21,22,23].

The pathological role played by astrocytes in ALS can be due to the loss of beneficial effects or to the gain of toxic functions, which are not mutually exclusive [24]. Astrocytes can affect MNs by transferring reactive oxygen/nitrogen species, complement components, chemokines, and cytokines [25,26]; modulating the microglial inflammatory response [27]; and reducing the efflux of lactate from oligodendrocytes for MN energy demand [24], all concurring to the development of Glu-mediated excitotoxicity [28,29,30].

A high percentage of sALS and fALS patients showed elevated levels of extracellular Glu in the motor cortex and spinal cord [31,32,33]. The mechanisms involved in abnormal Glu levels are complex and frequently intertwined by common cellular pathways altered in ALS, i.e., impairment of synaptic Glu clearance [28,34,35], excessive exocytotic and transporter-mediated Glu release [15,29,36,37,38,39], overactivation of excitatory Glu receptors and hyperexcitability [40,41,42], all contributing to neuronal death.

In this complex scenario, metabotropic glutamate receptors (mGluRs) play a subtle but crucial role [43,44,45]. In particular, the expression level of the group I mGluRs (mGluR1 and mGluR5), the only excitatory mGluR subtypes, was increased in reactive astrocytes in the brain and spinal cord of ALS patients [46] and SOD1^G93A^ mice [47]. SOD1^G93A^ astrocytes are highly vulnerable to Glu and die through an mGluR5-mediated mechanism [48,49]. Accordingly, we demonstrated in vivo that negatively modulating mGluR5 with a constitutive genetic ablation [30,50] or through a pharmacological blockade [51] significantly reduced glial activation and MN death, ameliorated the clinical progression and prolonged survival in SOD1^G93A^ ALS mice. Our evidence did not allow us to dissect the downstream molecular mechanisms after receptor inhibition or to clarify the specific contribution of different cell populations in the CNS. Assessing the impact of mGluR5 on the cell types involved in ALS would pave the way for potential cell-specific and more effective therapeutic interventions.

This study used our previously generated double mutant mice [50] carrying the SOD1^G93A^ mutation and a constitutive genetic mGluR5 downregulation (SOD1^G93A^mGluR5^+/−^). We explored the astrocyte phenotype by investigating Ca^2+^ overload, activation state, production and release of inflammatory factors, energy metabolism and neurotoxicity using spinal cord astrocytes cultured from adult late symptomatic SOD1^G93A^ or SOD1^G93A^mGluR5^+/−^ mice. Our results showed for the first time that reducing the mGluR5 expression dampens the astrocyte activation state, translating into reduced altered phenotype and neurotoxicity towards spinal MNs.

## 2. Materials and Methods

### 2.1. Animals

B6SJL-Tg (SOD1*G93A)1Gur mice expressing high copy number of mutant human SOD1 with a Gly93Ala substitution (SOD1^G93A^ mice [52] and B6.129-Grm5tm1Rod/J, carrying a null mutation for mGluR5 (Grm5^−/+^) [53]), were originally obtained from Jackson Laboratories (Bar Harbor, ME, USA), bred at the Animal Facility of the Pharmacology and Toxicology Unit, Department of Pharmacy in Genoa, and kept there until experiments were carried out. The SOD1^G93A^ mouse colony was maintained by crossing SOD1^G93A^ male mice with background-matched B6SJL wild-type (WT) females. Selective breeding maintained each transgene in the hemizygous state. Mice carrying the SOD1^G93A^ mutation were identified by analyzing tissue extracts from tail tips as previously described [50]. SOD1^G93A^ male mice were bred with Grm5^−/+^ females to generate SOD1^G93A^Grm5^−/+^ double-mutant mice carrying the Grm5^−/+^ heterozygous mutation and the SOD1*G93A transgene (responsible for the ALS pathological phenotype), as previously reported [50,54]. The breeding generated four littermates: WT, Grm5^−/+^, SOD1^G93A^ and SOD1^G93A^Grm5^−/+^ (Supplementary Figure S1). Grm5^−/+^ was identified by polymerase chain reaction (PCR) using specific primers, according to Bonifacino and colleagues [50]. Briefly, DNA was extracted from mice tails according to the manufacturer’s protocol (KAPA Mouse Genotyping Kits, Kapa Biosystems, Woburn, MA, USA) and amplified using 2 couples of primers. The first couple of primers (5′-CACATGCCAGGTGACATTAT-3′ and 5′-CCATGCTGGTTGCAGAGTAA-3′) amplified a product of 442 bp for the WT alleles. The second couple of primers (5′-CCCTAGAGCAAAGCATTGAGTT-3′ and 5′-GCCAGAGGCCACTTGTGTAG-3′) amplified a genomic fragment of 254 bp specific for the gene target insertion of the Grm5 null gene. All experiments were conducted on littermates derived from this last breeding. Mice were housed (6/7 per cage) at constant temperature (22 ± 1 °C) and relative humidity (50%) with a regular 12 h–12 h light cycle (light 7 a.m.–7 p.m.); food (type 4RF21 standard diet obtained from Mucedola, Settimo Milanese, Milan, Italy) and water were freely available. For experimental use, animals were sacrificed at a late stage of disease (around 120 days of age) and scored according to motor impairment severity [51,55]. The number of animals of each sex was balanced in all experimental groups to avoid bias due to intrinsic sex-related differences. All efforts were made to minimize animal suffering and to use only the number of animals necessary to produce reliable results. For ethical issues related to the use of animals for experimental studies, refer to the “Institutional Review Board Statement” section.

### 2.2. Mouse Spinal Cord Astrocyte Primary Cell Cultures

Spinal cord astrocyte primary cell cultures were prepared from adult 120-day-old WT, Grm5^−/+^, SOD1^G93A^ and SOD1^G93A^ Grm5^−/+^ mice as previously described [56] (Supplementary Figure S1). Briefly, late symptomatic SOD1^G93A^ mice and age-matched WT, Grm5^−/+^ and SOD1^G93A^Grm5^−/+^ animals were euthanized by cervical dislocation by trained personnel, and spinal cords were rapidly removed. The whole dissected spinal cord tissues were mechanically chopped with a scalpel, dispersed in complete Dulbecco’s Modified Eagle Medium (DMEM; Euroclone S.p.A, Milan, Italy, Cat# ECM0728L) containing 10% Fetal Bovine Serum (Euroclone, Cat# ECS0180L), 1% glutamine (Euroclone, Cat# ECB3004D) and 1% Penicillin/Streptomycin (Euroclone, Cat# ECB3001D), and further homogenized by using a P1000 pipette. Cell suspensions were seeded onto two 35 mm Petri dishes coated with poly-L-ornithine hydrochloride (1.5 µg/mL, Merck, Milan, Italy, Cat# P2533) and laminin (3 µg/mL; Merck, Milan, Italy, Cat# L2020). The preparations were maintained at 37 °C in a humidified 5% CO_2_ incubator for 4 days; then, the medium was replaced with fresh complete DMEM. After 7 days in vitro (DIV), cells were detached using Trypsin-EDTA 1X (Euroclone, Cat# ECB3052B) and replated until confluence. Astrocytes were seeded at the optimal density of 1 × 10^5^ cells in pre-coated 6-well plates for Western blot (WB), RT-qPCR, biochemical assays, ELISA assays and glutamate quantification, or in pre-coated 35 mm dishes for astrocyte-MN co-cultures. Astrocytes were seeded at a density of 3 × 10^4^ cells/well in 24-well plates containing 12 mm diameter pre-coated glass coverslips for immunofluorescence (IF) experiments. The culture medium was systematically replaced with fresh medium every two days. Astrocytes were used for experiments after 20 DIV. On the day of the experiment, the culture medium was removed, and cells were washed with PBS. The astrocyte purity was checked by flow cytometry and immunofluorescence, as previously described (Supplementary Figure S1) [56,57].

### 2.3. Western Blot

WT, Grm5^−/+^, SOD1^G93A^ and SOD1^G93A^Grm5^−/+^ astrocytes were prepared for WB as previously described by Provenzano and colleagues [56] with minor modifications. Briefly, after detaching with Trypsin-EDTA 1X and washing in PBS, astrocytes were centrifuged at 17,000× *g* for 5 min at 4 °C. PBS was removed, and cell pellets resuspended in Milli-Q water plus protease inhibitor (Merck, Milan, Italy, Cat# P8340) and sonicated in ice twice for 10 s, with a 30 s break, to prevent the mixture from warming, using the Microson XL Model DU-2000 (Mis-onix Inc., Farmingdale, NY, USA). Total protein content was estimated with the Bradford method [58]. Then, 30 μg samples of total proteins were separated by SDS-polyacrylamide gel electrophoresis using 4–20% precast gels (Bio-Rad, Milan, Italy, Cat# 4568094) and transferred to nitrocellulose membranes (NC, Bio-Rad Laboratories) by electroblotting at 400 mA for 2 h in Tris-glycine buffer (50 mM Tris, 380 mM glycine) plus 20% methanol. The membrane was blocked using 5% skimmed-milk solution for 1 h at RT and then incubated overnight at 4 °C with primary antibodies properly diluted in 5% skimmed-milk solution (Supplementary Table S1). Afterward, membranes were washed in 0.15% Tween 20 (Merck, Milan, Italy, Cat#P7949-500 mL) in PBS (PBSt), incubated for 1 h at RT with specific HRP secondary antibodies (Supplementary Table S1) and developed using Clarity Western ECL Substrate (Bio-Rad Laboratories). Bands were analyzed for density by using the Alliance 6.7 WL 20 M enhanced chemiluminescence system and UV1D software (latest version v10, UVITEC, Cambridge, UK). Each band was converted into a densitometric trace allowing intensity determination, signals were normalized to the signal of GAPDH and results were expressed as relative optical density (R.O.D.).

### 2.4. Immunofluorescence Experiments

WT, Grm5^−/+^, SOD1^G93A^ and SOD1^G93A^Grm5^−/+^ astrocytes were seeded on 12 mm diameter glass coverslips at the bottom of 24-well plates and processed for IF analyses as previously described [56]. Briefly, astrocytes were fixed with 4% PFA (Merck, Milan, Italy, Cat# 47608) and permeabilized with methanol for 5 min at −20 °C. BSA diluted in PBS (0.5%) was applied for 15 min at RT to arrest the process and saturate the unspecific binding sites. Primary antibodies were properly diluted in 3% PBS-BSA blocking solution and incubated overnight at 4 °C (Supplementary Table S2). In the experiments studying the plasma membrane expression of mGluR5 co-localized with lectin, spinal cord astrocyte cultures were fixed with 4% PFA and directly exposed to the blocking solution (0.5% BSA in PBS), avoiding permeabilization, before incubation with primary antibodies. Then, cells were washed with 0.5% PBS-BSA and incubated for 1 h at RT with secondary antibodies diluted 1:3000 in 3% PBS-BSA (Supplementary Table S2). Astrocytes were washed in PBS, and the coverslips were assembled on a microscopy glass slide through Fluoroshield with DAPI (Merck, Milan, Italy, Cat# F6057). Fluorescence image (512 × 512 × 8 bit) acquisition was performed using a three-channel TCS SP5 laser-scanning confocal microscope (Leica, Wetzlar, Germany) equipped with 458, 476, 488, 514, 543 and 633 nm excitation lines, through a plan-apochromatic oil immersion objective 63× (1.4 NA). Light collection was optimized according to the combination of the chosen fluorochromes, and sequential channel acquisition was performed to avoid crosstalk. The Leica “LAS AF” software package (latest version 4.0) was used for image acquisition. The quantitative analyses were performed, using Fiji ImageJ free software (latest version 2.9.0), as previously described [30,37,56], by calculating the co-localization coefficients, according to Manders and Costes [59,60], thus allowing a direct quantitative correlation between the intensity of the co-localization of the protein of interest and the stable housekeeping protein 3-phosphate dehydrogenase glyceraldehyde (GAPDH). GAPDH fluorescent signal intensity was determined by selecting the ROI of interest and measuring the area, integrated density and mean grey value. The corrected total cell fluorescence (CTCF; arbitrary units) was quantified as integrated density (area of the selected cell × mean fluorescence of background readings (https://theolb.readthedocs.io/en/latest/imaging/measuring-cell-fluorescence-using-imagej.html; accessed on 1 July 2023), as previously described [61,62]. The GAPDH fluorescent intensity was comparable in the different experiments (Supplementary Table S3). At least 3 independent experiments, run in triplicate (i.e., 3 wells per experiment), were performed. Each acquired image included 6 to 12 cells homogenously distributed, thus resulting in 50 to 100 assessed cells for each sample in each experiment.

### 2.5. RT-qPCR

WT, Grm5^−/+^, SOD1^G93A^ and SOD1^G93A^ Grm5^−/+^ astrocytes were detached with Trypsin-EDTA 1X (Euroclone, S.p.A, Milan, Italy, Cat# ECB3052B) and centrifuged at 700× *g* for 5 min at RT. The cell pellet was washed in PBS, centrifuged at 17,000× *g* for 5 min at 4 °C, and lysed in 500 µL TRIzol (Thermo Fisher Scientific, Monza, Italy, Cat# 15596026). RNA was purified using the ReliaPrep RNA Cell Miniprep System (Promega, Milan, Italy, Cat# Z6010) and quantified by optical density (NanoDrop ND-2000 Spectrophotometer, NanoDrop Technologies, Thermo Fisher Scientific, Monza, Italy). cDNA was prepared from 0.6 μg RNA using the iScript Reverse Transcription Supermix for RT-qPCR Kit (Bio-Rad, Cat# 1708840). Real-time PCR was performed in an IQ5 Multicolor Real-Time PCR Detection System (Bio-Rad Laboratories, Hercules, CA, USA) using iQ SYBR Green Supermix (Bio-Rad Laboratories, Cat# 1708882), as previously described [63]. Briefly, PCR amplifications were performed in triplicate using 1:2 diluted cDNA in a 25 μL final reaction mixture. Mouse GAPDH cDNA was used as a housekeeping gene. The following primers were used: Grm5 (Grm5_F: 5′-AGCAAGTGATCAGAAAGACTCG-3′ and Grm5_R: 5′-GTCACAGACTGCAGCAGAGC-3′); GAPDH (GAPDH_F: 5′-ATTGTCAGCAATGCATCCTG-3′ and GAPDH_R: 5′-ATGGACTGTGGTCATGAGCC-3′). RT-qPCR conditions were the following: 10 s at 95 °C for 10 s, 30 s at 60 °C, 30 s at 72 °C, for 40 cycles. The mRNA expression was calculated using the ΔΔCt method [64], normalizing to GAPDH. Data from mGluR5 ASO- and control ASO-treated mice were compared to WT, normalized to 100%.

### 2.6. Cytosolic Calcium Concentration

Intracellular calcium concentration ([Ca^2+^]_i_) was determined in WT, SOD1^G93A^ and SOD1^G93A^Grm5^−/+^ astrocytes seeded on 12 mm diameter coated coverslips inside 24-well plates, using the fluorescent dye Fura-2/AM [65]. After 20 DIV astrocytes were incubated for 40 min at 37 °C in complete DMEM supplemented with 10 µM of Fura-2/AM previously solubilized in 0.5% Dimethyl-sulfoxide (DMSO, Merck, Milan, Italy, Cat# D2650), astrocytes incubated with complete DMEM supplemented only with 0.5% DMSO were used to measure the auto-fluorescence. After incubation, cells were washed in PBS with 0.9 mM CaCl_2_ × 2 H_2_O (Merck, Milan, Italy, Cat# D8662-500ML) to remove the excess of Fura-2/AM. The same solution was used to measure the [Ca^2+^]_i._ The [Ca^2+^]_i_ measurements were performed at 37 °C using an RF-5301PC dual-wavelength spectrofluorophotometer (Shimadzu Corporation, Milan, Italy) by alternating the excitation wavelengths of 340 and 380 nm. Fluorescent emission was monitored at 510 nm. Basal fluorescence was recorded for 1 min, and then astrocytes were exposed to 30 µM 3,5-DHPG, a group I mGluR agonist. Calibration of the fluorescent signal was performed at the end of each measure by adding 10 µM Ionomycin to obtain the maximum fluorescence signal (F_max_), followed by 10 mM EDTA adjusted to pH 8.0 and buffered with 3 mM Tris, to obtain the minimum fluorescence signal (F_min_). [Ca^2+^]_i_ was calculated using the equation of Grynkiewicz and colleagues [65] using a K_D_ of 224 nM for the Ca^2+^-Fura-2 complex. Measures were performed within 2 h from astrocyte labeling.

### 2.7. In Vitro Treatments with a Specific mGluR5 Antisense Oligonucleotide and a Selective mGluR5 Negative Allosteric Modulator

Antisense oligonucleotide. Antisense oligonucleotides (ASOs) used in this study were chemically modified single-stranded nucleic acids of 20 nucleotides in length with 5 2′ -O-methoxyethyl modified nucleotides at each end and 10 DNA nucleotides in the centre. The mouse-specific mGluR5 (5′-CTTGTCACTCAAATCCATGC-3′) binds to mGluR5 pre-mRNA via Watson–Crick hybridization and degrades it by recruiting RNaseH1. A non-targeting ASO (5′-CCTATAGGACTATCCAGGAA-3′) was used as a negative control. Ionis Pharmaceuticals designed and developed these ASOs as previously described [66]. For in vitro treatment, spinal cord astrocytes cultured from WT and SOD1^G93A^ adult mice were seeded at a density of 2–3 × 10^4^ cells/well in 24-well plates containing 12 mm diameter pre-coated glass coverslips. SOD1^G93A^ astrocytes were treated with the mGluR5 ASO (10 µM) for 48 h. Control SOD1^G93A^ astrocytes were treated with 10 µM control ASO. After 48 h of mGluR5 or control ASO in vitro exposure, cells were washed in PBS, and complete DMEM was added for an additional 5 days. After 7 days, WT and SOD1^G93A^ astrocytes were detached and collected for RT-qPCR analysis to verify the mGluR5 mRNA reduction. Samples were fixed in 4% PFA for IF analysis.

Negative allosteric modulator. The highly selective mGluR5 negative allosteric modulator 2-chloro-4-((2,5-dimethyl-1-(4-(trifluoromethoxy) phenyl)-1H-imidazol-4-yl)ethynyl)pyridine (CTEP) [67] was synthetized, purified and kindly provided by Prof. Alfei [68] (Organic Chemistry Unit of the Department of Pharmacy, University of Genoa), as previously described [51]. For the in vitro treatment, spinal cord astrocytes cultured from WT and SOD1^G93A^ adult mice were seeded at a density of 2-3 × 10^4^ cells/well in 24-well plates containing 12 mm diameter pre-coated glass coverslips. SOD1^G93A^ astrocytes were treated for 7 days with 0.1 µM CTEP dissolved in DMSO and complete DMEM. DMSO alone in DMEM was used as a control. Culture media containing CTEP or DMSO were replaced every 48 h. On day 7, cells were washed twice with PBS 1X, and complete DMEM was added for an additional 24 h. Then, astrocytes were fixed with 4% PFA for IF analysis.

### 2.8. Evaluation of the Energetic Profile

Oxygen consumption rate. Oxygen consumption rate (OCR) was evaluated in WT, SOD1^G93A^ and SOD1^G93A^Grm5^−/+^ astrocytes using a thermostatically controlled (37 °C) oxygraph apparatus equipped with an amperometric electrode (Unisense-Microrespiration, Unisense A/S, Aarhus, Denmark). In each experiment, 1 × 10^5^ cells were used. Astrocytes were permeabilized with 0.03% digitonin for 10 min and suspended in the respiration medium containing 137 mM NaCl, 5 mM KCl, 0.7 mM KH_2_PO_4_, 25 mM Tris–HCl pH 7.4 and 25 mg/mL ampicillin. The respiring substrates pyruvate (10 mM) and malate (5 mM) or succinate (20 mM) were added to stimulate Complexes I, III, and IV or II, III, and IV, respectively. Then, 0.1 mM ADP was added after the addition of the respiratory substrates. The respiratory rate was expressed as nmol atomic oxygen/min/10^6^ cells [69].

F_o_-F_1_ ATP synthase activity. F_o_-F_1_ ATP synthase activity was evaluated in WT, SOD1^G93A^ and SOD1^G93A^Grm5^−/+^ astrocytes. First, 1 × 10^5^ cells were incubated for 10 min at 37 °C in a medium containing 10 mM Tris-HCl pH 7.4, 100 mM KCl, 5 mM KH_2_PO_4_, 1 mM EGTA, 2.5 mM EDTA, 5 mM MgCl_2_, 0.6 mM ouabain, 0.3 mM P1, P5-Di (adenosine-5′) pentaphosphate and 25 mg/mL ampicillin. ATP synthesis was induced by pyruvate (10 mM) and malate (5 mM) or succinate (20 mM). The reaction was initiated by adding 0.1 mM ADP and monitored every 30 s for 2 min, in a luminometer (GloMax 20/20n Luminometer, Promega Italia, Milano, Italy), by the luciferin/luciferase chemiluminescence method, with ATP standard solutions between 10^−8^ M and 10^−5^ M (luciferin/luciferase ATP bioluminescence assay kit CLSII, Roche, Basel, Switzerland). Data were expressed as nmol ATP/min/10^6^ cells [69].

Oxidative phosphorylation efficiency. The oxidative phosphorylation (OxPhos) efficiency was calculated as the ratio between the concentration of ATP produced and the amount of oxygen consumed, defining the P/O ratio. When oxygen consumption is completely devoted to energy production, the P/O ratio should be around 2.5 and 1.5 after pyruvate and malate or succinate addition, respectively [70].

Glucose consumption. To evaluate glucose consumption, the glucose content in the culture medium of WT, SOD1^G93A^ and SOD1^G93A^Grm5^−/+^ astrocytes was evaluated in a double beam spectrophotometer (UNICAM UV2, Analytical S.n.c., Milan, Italy) by the hexokinase (HK) and glucose 6 phosphate dehydrogenase (G6PD) coupling method, following the reduction of NADP at 340 nm. The NADH/NADPH molar extinction coefficient was considered 6.22 × 10^−3^ M/cm at 340 nm [71]. The assay medium contained 100 mM Tris-HCl pH 7.4, 2 mM ATP, 10 mM NADP, 2 mM MgCl_2_, 2 IU of hexokinase and 2 IU of glucose 6-phopshate dehydrogenase. Data were normalized to the cell number and expressed as mM glucose consumed/10^6^ cells [72].

Lactate dehydrogenase activity and lactate release assay. Lactate dehydrogenase (LDH, EC 1.1.1.27) activity was measured by determining NADH oxidation at 340 nm. The NADH molar extinction coefficient was considered 6.22 × 10^−3^ M/cm at 340 nm. The reaction mixture contained 100 mM Tris–HCl pH 9.5 mM pyruvate and 0.2 mM NADH. Enzymatic activity was expressed as IU/mg of total protein [73]. Lactate concentration was assayed spectrophotometrically in the culture medium of WT, SOD1^G93A^ and SOD1^G93A^Grm5^−/+^ astrocytes, following the reduction of NAD^+^ at 340 nm [71]. The assay medium contained 100 mM Tris-HCl at pH 8.5 mM NAD^+^ and 1 IU/mL LDH. Data were normalized to the cell number and expressed as mM released lactate/10^6^ cells [72].

### 2.9. Enzyme-Linked Immunosorbent Assay

The culture medium of 20 DIV derived from WT, SOD1^G93A^ and SOD1^G93A^Grm5^−/+^ astrocytes was substituted and collected 24 h after replacement. Media were filtered with a 0.22 µm sterile filter before analyses. Then, TNF-α, IL-1β and IL-6 contents were measured by specific enzyme-linked immunosorbent assay (ELISA) kits (R&D Systems, Cat# DY401, DY410 and DY406) according to the manufacturer’s protocol.

### 2.10. Glutamate Quantification

The culture medium of 20 DIV derived from WT, SOD1^G93A^ and SOD1^G93A^Grm5^−/+^ astrocytes was substituted with a fresh physiological medium having the following composition (mM): NaCl, 140; KCl, 3; MgSO4, 1.2; NaH_2_PO_4_, 1.2; NaHCO_3_, 5; CaCl_2_, 1.2; 4-(2-hydroxyethyl)-1-piperazineethanesulfonic acid (Hepes), 10; glucose, 10; pH 7.4. The media were collected after 4 h and filtered with a 0.22 µm sterile filter before analyses. Endogenous Glu content in the cultured media was measured by high-performance liquid chromatography (HPLC, Alliance 2095 module with remote control by the Millenium 32 Chromatography Empower 3 Manager Software; Waters Italia s.p.a, Sesto San Giovanni, Italy) after OPT (phthaldialdehyde) buffer derivatization and fluorometric detection (Shimadzu RF-10AXL; excitation 350 nm; emission 450 nm) as previously described [74,75].

### 2.11. Spinal Cord Motor Neuron Preparation and Co-Cultures with Astrocytes

Motor neurons (MNs) were prepared from the spinal cord of WT and SOD1^G93A^ E13.5 mouse embryos as previously described with minor modifications [56,76,77]. Briefly, spinal cords were dissected under microscopy (Carl Zeiss 475110-9902 Microscope, Milan, Italy). Meninges and dorsal root ganglia were removed. The ventral spinal cord was cut into small pieces and digested in 0.5% trypsin (Merck, Milan, Italy, Cat# T4799) in Hank’s Balance Salt Solution (HBSS, Euroclone, S.p.A, Milan, Italy, Cat# ECM0507L) for 20 min at 37 °C. The trypsin solution was replaced with a mix of 0.02 mg/mL deoxyribonuclease I (DNAse; Merck, Milan, Italy, Cat#DN25) and 0.4% BSA (Sigma-Aldrich, Cat# A3311) in Leibovitz-15 medium (Merck, Milan, Italy, Cat# L5520) and gently triturated. The tissue homogenate was stratified on a 6.2% OptiPrep (Merck, Milan, Italy, Cat# D1556) cushion and centrifuged at 500× *g* for 15 min at RT. The MN-enriched cell population was resuspended in an MN complete medium, composed of neurobasal medium (Thermo Fisher Scientific, Monza, Italy, Cat# 21103-049), 2% B27 supplement (ThermoFisher Scientific, Monza, Italy, Cat# 17504044), 2% horse serum (Thermo Fisher Scientific, Monza, Italy, Cat# 16050130), 0.5 mM stable L-Glutamine (Thermo Fisher Scientific, Monza, Italy, Cat#35050038), 25 µM Mercapto ethanol (Merck, Milan, Italy, Cat# M6250), 10 ng/mL ciliary neurotrophic factor (CNTF; Merck, Milan, Italy, Cat# C3835), 100 pg/mL glial-derived neurotrophic factor (GDNF; Merck, Milan, Italy, Cat# G1401) and 5 µg/mL Penicillin/Streptomycin. MN suspensions were layered on the top of a 4% BSA gradient containing 20 µL of 1 mg/mL DNAse and centrifuged at 75× *g* for 20 min to remove Optiprep impurity. Then, the pellet was suspended in 1 mL of MN medium containing 50 µL of Chick Embryo Extract (US Biological, MA, US, Cat# C3999). Then, 5 × 10^4^ MNs were seeded in a 35 mm Petri dish on a confluent layer of spinal cord astrocytes prepared from 120-day-old WT, SOD1^G93A^ or SOD1^G93A^Grm5^−/+^ mice. MN purity was assessed as previously described [56] (Supplementary Figure S2). During culturing, the medium was replaced with fresh MN complete medium every 48 h. MN viability was assessed as previously described [56]. MNs were counted in an area equal to 1 cm^2^ (using a 10 mm × 10 mm grid pre-designed at the bottom of the Petri dish). Starting from day 4 after seeding, the number of viable MNs in the predesigned 1 cm^2^ grid was recorded three times a week for 14 days. The number of surviving MNs at each time point was calculated as % of the total number of MNs, counted in the same 1 cm^2^ square area of the respective dish, at day 4 of co-culture, as the starting day of cell counting (reported as 100% of total MNs).

### 2.12. Statistics

GraphPad Prism Software (Version 9, license code GP9-2314983-RATL-05225; 225 Franklin Street. Fl. 26, Boston, MA 02110; RRID:SCR_002798) was used for statistical analysis and for figure plots. All experiments were performed with a minimum of three independent biological replicates. The number of biological and technical replicates, statistical tests used and *p* values are reported in the figure legends. The threshold for statistical significance (*p*) was set at *p* < 0.05. Data are always presented as mean ± standard error of the mean (SEM).

## 3. Results

### 3.1. Genetic mGluR5 Ablation Reduces the Receptor Expression in SOD1^G93A^ Spinal Cord Astrocytes

We investigated the total mGluR5 protein expression by WB and the plasma membrane localization by IF confocal microscopy using an antibody selective for an external epitope of the mGluR5 and quantified the co-localization of mGluR5 with lectin as a marker of the plasma membrane. Experiments were performed with primary spinal cord astrocyte cultures of WT, Grm5^−/+^, SOD1^G93A^, and SOD1^G93A^Grm5^−/+^ 120-day-old mice to validate the genetic downregulation of mGluR5 in SOD1^G93A^ astrocytes.

**Figure 1:**
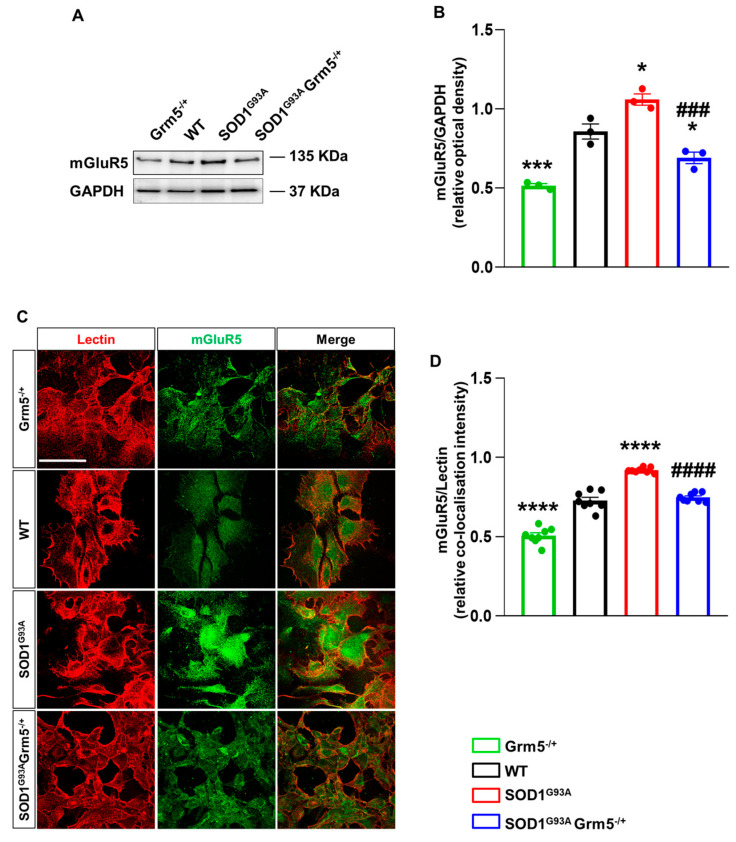
mGluR5 expression in spinal cord astrocytes cultured from adult Grm5^−/+^, WT, SOD1^G93A^, and SOD1^G93A^Grm5^−/+^ mice. (**A**) Representative Western blot (WB) showing immunoreactive mGluR5 bands. (**B**) Quantification of WB mGluR5 densitometric signals. Protein band density was normalized to GAPDH, as a housekeeping protein. Data are means ± s.e.m of *n* = 3 independent experiments. * *p* < 0.05 and *** *p* < 0.001 vs. WT astrocytes; ### *p* < 0.001 vs. SOD1^G93A^ astrocytes (F_(3, 8)_ = 43.43; one-way ANOVA followed by Tukey’s multi-comparison test). (**C**) Representative confocal microscopy immunocytochemical images of mGluR5 (green fluorescence) and lectin (red fluorescence). Scale bar 100 µm. Grm5^−/+^, WT, SOD1^G93A^, and SOD1^G93A^Grm5^−/+^ spinal cord astrocytes were fixed with paraformaldehyde and incubated with the primary antibodies for mGluR5 and lectin, without membrane permeabilization, and subsequently with fluorescent secondary antibodies. Images were acquired by confocal microscopy. (**D**) Quantitative representation of mGluR5 expression was calculated as the relative fluorescence intensity of mGluR5 co-localized with lectin, that labels the plasma membrane. Data are means ± s.e.m of *n* = 8 independent experiments. **** *p* < 0.0001 vs. WT astrocytes; #### *p* < 0.0001 vs. SOD1^G93A^ astrocytes (F_(3,28)_ = 134.3; one-way ANOVA followed by Tukey’s multi-comparison test).

Our WB analysis showed overexpression of mGluR5 in SOD1^G93A^ compared to WT spinal cord astrocytes, in line with previously published data [46,48,78]. mGluR5 overexpression was effectively hampered in SOD1^G93A^Grm5^−/+^ vs. SOD1^G93A^ astrocytes and halved in Grm5^−/+^ vs. WT astrocytes (Figure 1A,B). The plasma membrane mGluR5 expression increased significantly in astrocytes cultured from SOD1^G93A^ mice in comparison to WT and Grm5^−/+^ astrocytes (Figure 1C,D). The mGluR5 plasma membrane overexpression was normalized in SOD1^G93A^Grm5^−/+^ spinal cord astrocytes (Figure 1D). RT-qPCR experiments confirmed the protein analysis since the heterozygous genetic ablation of the *Grm5* gene encoding for mGluR5 reduced the mGluR5 transcript in adult spinal cord astrocyte cultures from the Grm5^−/+^ and SOD1^G93A^Grm5^−/+^ mice vs. WT and SOD1^G93A^ astrocytes, respectively (Supplementary Figure S3).

### 3.2. Downregulation of mGluR5 Reduces the Elevated Cytoplasmic Ca^2+^ Levels in SOD1^G93A^ Spinal Cord Astrocytes

**Figure 2:**
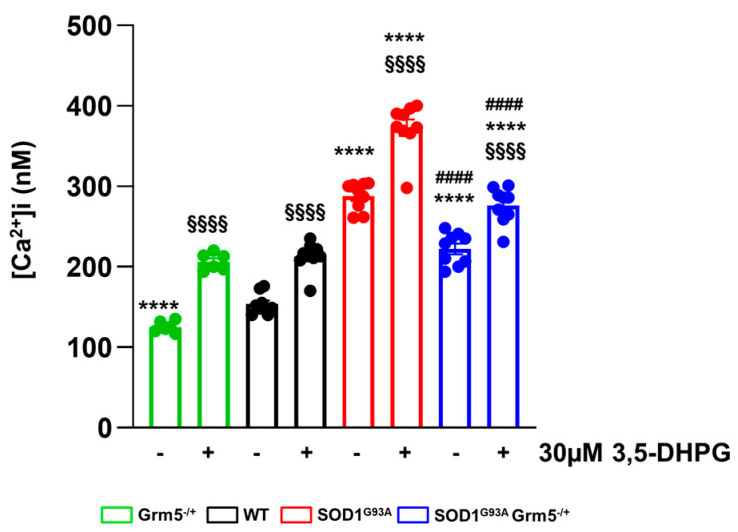
Ca^2+^ concentration [Ca^2+^]i under basal and stimulated conditions in spinal cord astrocytes cultured from adult WT, Grm5^−/+^, SOD1^G93A^, and SOD1^G93A^Grm5^−/+^ mice. Astrocytes were labeled with the fluorescent dye Fura2-AM. [Ca^2+^]_i_ was measured by ratiometric spectrofluorophotometry in basal conditions and after stimulation with the selective group I metabotropic glutamate receptor agonist 3,5-DHPG (30 μM). Results are means ± s.e.m of *n* = 6–9 independent experiments and are expressed as nanomolar (nM) Ca^2+^ concentration [Ca^2+^]i. §§§§ *p* < 0.0001 stimulated vs. the respective basal conditions; **** *p* < 0.0001 vs. WT astrocytes, under basal or stimulated conditions; #### *p* < 0.0001 vs. SOD1^G93A^ astrocytes under basal or stimulated conditions (F_(1,58)_ = 207.2 and F_(3,58)_ = 240.5; two-way ANOVA followed by Tukey’s multi-comparison test).

Abnormal intracellular calcium signaling is a key player sustaining astrocyte-mediated neurotoxicity in ALS [48,79]. mGluR5 activation can trigger intracellular pathways inducing [Ca^2+^]_i_ oscillations resulting in pathological conditions. Therefore, we investigated the effect of partial mGluR5 genetic deletion on [Ca^2+^]_i_ in our experimental conditions. The [Ca^2+^]_i_ was measured in WT, Grm5^−/+^, SOD1^G93A^, and SOD1^G93A^Grm5^−/+^ spinal cord astrocytes using the fluorescent dye FURA-2/AM under basal condition and after a stimulus with 30 µM 3,5-DHPG. The [Ca^2+^]_i_ was dramatically increased in SOD1^G93A^ compared to WT astrocytes under basal conditions. The elevated basal [Ca^2+^]_i_ in SOD1^G93A^ astrocytes was significantly reduced in spinal cord astrocytes cultured from double mutant SOD1^G93A^Grm5^−/+^ mice, although [Ca^2+^]_i_ did not return to the WT astrocyte level (Figure 2). Exposure of primary astrocyte cultures to the selective group I mGluR agonist 3,5-DHPG (30 µM) significantly increased [Ca^2+^]_i_ over the basal level in all astrocyte genotypes. Of note, the 3,5-DHPG-stimulated [Ca^2+^]_i_ was significantly reduced in SOD1^G93A^Grm5^−/+^ astrocytes compared to SOD1^G93A^ astrocytes (Figure 2).

These results indicate that the genetic downregulation of mGluR5 effectively translates into a constitutive reduction in the [Ca^2+^]_i_, which is higher in SOD1^G93A^ astrocytes.

### 3.3. Downregulation of mGluR5 Reduces the Reactive Phenotype of SOD1^G93A^ Spinal Cord Astrocytes

The altered astrocyte activation that sustains a neuroinflammatory and neurotoxic phenotype represents a key pathological feature of ALS [48,80,81]. We first investigated the expression of GFAP, vimentin and S100β, three proteins related to the proliferative and pathological reactivity of ALS astrocytes [82,83,84,85] in WT, Grm5^−/+^, SOD1^G93A^, and SOD1^G93A^Grm5^−/+^ astrocytes cultured from the spinal cord of adult mice, by WB and immunocytochemistry.

**Figure 3:**
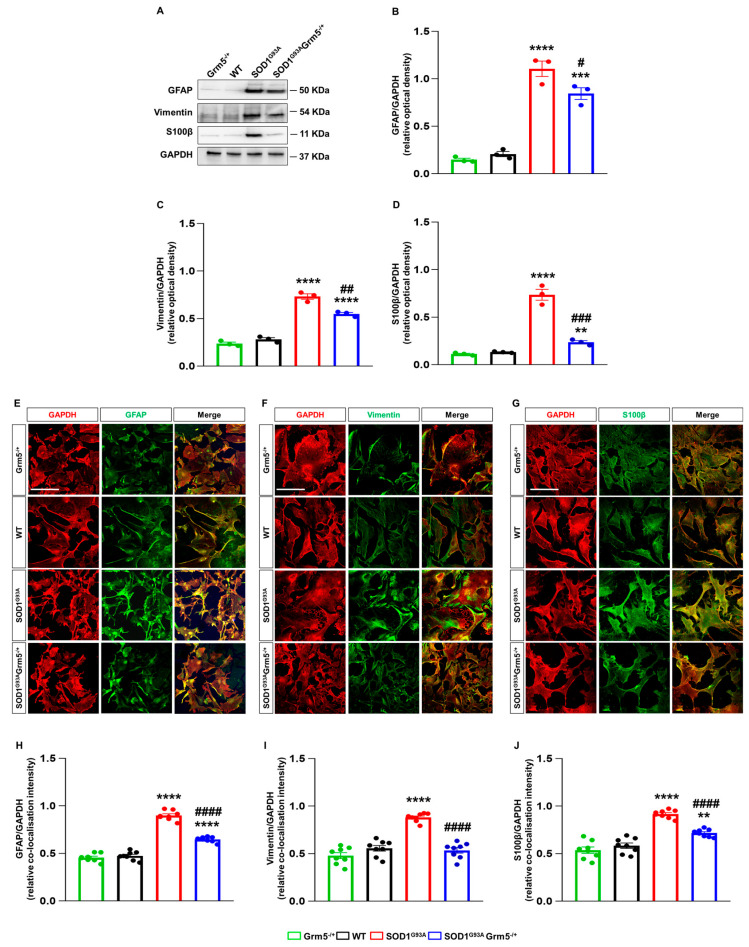
Expression and cellular localization of the astrocyte activation markers GFAP, vimentin, and S100β in spinal cord astrocytes cultured from adult WT, Grm5^−/+^, SOD1^G93A^, and SOD1^G93A^Grm5^−/+^ mice. (**A**) Representative Western blots (WBs) for GFAP, vimentin and S100β (**B**–**D**). Densitometric quantification of WB signals of (**B**) GFAP, (**C**) vimentin and (**D**) S100β. Protein band density was normalized to GAPDH as a housekeeping protein. Data are means ± s.e.m of *n* = 3 independent experiments. ** *p* < 0.01, *** *p* < 0.001 and **** *p* < 0.0001 vs. WT astrocytes; # *p* < 0.05, ## *p* < 0.01 and ### *p* < 0.001 vs. SOD1^G93A^ astrocytes, (F_(3,8)_ = 77.99, F_(3,8)_ = 131.5 and F_(3,8)_ = 80.76 for GFAP, vimentin and S100β, respectively; one-way ANOVA followed by Tukey’s multi-comparison test). (**E**–**G**) Representative confocal microscopy immunocytochemical images of GFAP (**E**), vimentin (**F**) and S100β (**G**) (green fluorescence) and GAPDH (red fluorescence). Grm5^−/+^, WT, SOD1^G93A^ and SOD1^G93A^Grm5^−/+^ spinal cord astrocytes were fixed, permeabilized and incubated with appropriate primary and fluorescent secondary antibodies. Images were acquired by confocal microscopy. Scale bar: 100 µm. Quantitative representation of GFAP (**H**), vimentin (**I**), and S100β (**J**) expression, calculated as the relative fluorescence intensity of the protein of interest co-localized with the reference protein GAPDH. Data are means ± s.e.m of *n* = 7–8 independent experiments. ** *p* < 0.01 and **** *p* < 0.0001 vs. WT astrocytes; #### *p* < 0.0001 vs. SOD1^G93A^ astrocytes (F_(3,28)_ = 190.5, F_(3,28)_ = 44.80 and F_(3,28)_= 49.33 for GFAP, vimentin and S100β, respectively; one-way ANOVA followed by Tukey’s multi-comparison test).

WB showed a significantly higher expression of all the markers in SOD1^G93A^ compared to WT astrocytes (Figure 3A–D). In Grm5^−/+^ astrocytes, the levels of GFAP, vimentin and S100β expression were comparable to those in WT astrocytes (Figure 3A–D), while the partial mGluR5 deletion significantly reduced the overexpression of GFAP, vimentin and S100β.

Confocal microscopy experiments confirmed the above results, showing that the expression levels of astrogliosis markers GFAP, vimentin and S100β were increased in SOD1^G93A^ and recovered, at least in part, in SOD1^G93A^Grm5^−/+^ spinal cord astrocytes (Figure 3E–J).

Of note, IF confocal microscopy experiments revealed that the constitutive genetic mGluR5 downregulation also reduced the cytoplasmic accumulation of misfolded SOD1 in adult spinal cord SOD1^G93A^ Grm5^−/+^ astrocytes (Supplementary Figure S4). This result unveils a possible correlation between the mGluR5 receptor downregulation and the clearance of SOD1 aggregates in ALS, also in line with literature evidence showing that mGluR5 antagonism increases autophagy and prevents disease progression in a Huntington’s disease mouse model [86].

Since all the data obtained until now showed no significant difference between WT and Grm5^−/+^astrocytes, we omitted this control in the next analyses.

### 3.4. Reducing mGluR5 In Vitro by Antisense Oligonucleotide and Negative Allosteric Modulation Mimics the In Vivo Genetic Downregulation

The genetic mGluR5 downregulation in vivo is a chronic situation of mGluR5 deprivation during the animals’ lifetime. To verify the effects of acute mGluR5 downregulation, more closely resembling a possible therapeutic approach in patients after the diagnosis, we exploited genetic and pharmacological strategies. We exposed astrocytes cultured from the spinal cord of late symptomatic SOD1^G93A^ mice to a specific antisense oligonucleotide (ASO) anti-mGluR5 and to the negative allosteric mGluR5 modulator (NAM) CTEP [67].

**Figure 4:**
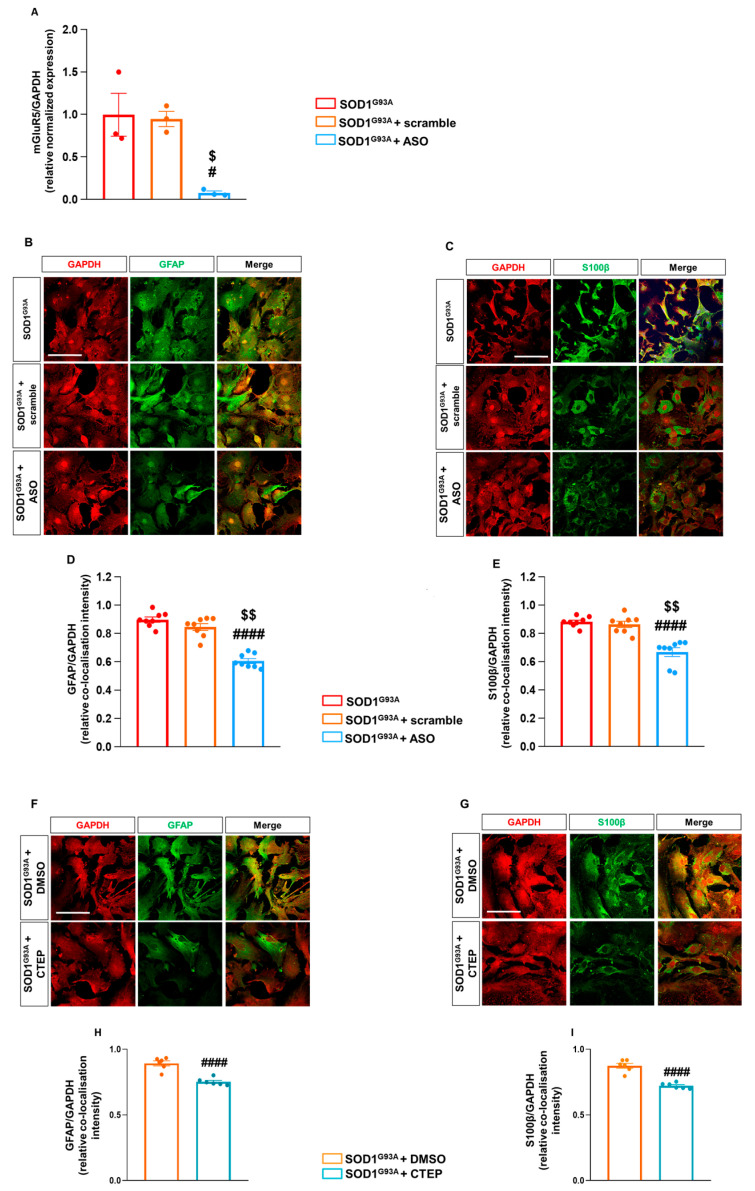
Effect of in vitro exposure to an mGluR5 antisense oligonucleotide and the mGluR5 selective negative allosteric modulator CTEP in spinal cord astrocytes cultured from adult SOD1^G93A^ mice. (**A**) RT-qPCR quantitative analyses for *Grm5* expression in untreated, scramble- or ASO-treated (20 µM) SOD1^G93A^ spinal cord astrocytes. Data are means ± s.e.m of *n* = 3 independent experiments run in triplicate. # *p* < 0.05 vs. untreated SOD1^G93A^ astrocytes; $ *p* < 0.05 vs. scramble-treated SOD1^G93A^ astrocytes (F_(2,6)_ = 11.15; one-way ANOVA followed by Tukey’s multi-comparison test). (**B**,**C**) Representative confocal microscopy immunocytochemical images of (**B**) GFAP (green fluorescence) and GAPDH (red fluorescence) and (**C**) S100β (green fluorescence) and GAPDH (red fluorescence) in untreated, scramble- and ASO-treated SOD1^G93A^ astrocytes. Scale bar: 100 µm. Astrocytes were labeled with appropriate primary and fluorescent secondary antibodies, and the images were acquired by confocal microscopy. Quantitative representation of GFAP (**D**) and S100β (**E**) expression, calculated as the relative fluorescence intensity of the protein of interest co-localized with the reference protein GAPDH. Data are means ± s.e.m of *n* = 8 independent experiments. #### *p* < 0.0001 vs. untreated SOD1^G93A^ astrocytes and $$ *p* < 0.0001 vs. scramble-treated SOD1^G93A^ astrocytes (F_(2,21)_ = 24.39 and F_(2,21)_ = 60.46 for GFAP and S100β, respectively; one-way ANOVA followed by Tukey’s multi-comparison test). (**F**,**G**) Representative confocal microscopy immunocytochemical images of (**F**) GFAP (green fluorescence) and GAPDH (red fluorescence) and (**G**) S100β (green fluorescence) and GAPDH (red fluorescence) in SOD1^G93A^ astrocytes exposed for 7 days to DMSO or CTEP (100 nM). Scale bar: 100 µm. (**H**,**I**) Quantitative representation of GFAP (**H**) and S100β (**I**) protein expression, calculated as described above. Data are means ± s.e.m of *n* = 6 independent experiments. ^####^ *p* < 0.0001 vs. SOD1^G93A^ astrocytes treated with DMSO (F_(2,15)_ = 209.8 and F_(2,15)_ = 234.2 for GFAP and S100β, respectively; one-way ANOVA followed by Tukey’s multi-comparison test).

Spinal cord astrocytes from late symptomatic SOD1^G93A^ mice were exposed to the anti-mGluR5 ASO (20 µM) or control ASO for 48 h, and the astrocyte mGluR5 expression and the modulation of reactive astrogliosis were investigated. Quantitative RT-qPCR demonstrated that exposure of SOD1^G93A^ astrocytes to the ASO abolished the mGluR5-encoding mRNA expression compared to untreated or scramble-treated SOD1^G93A^ astrocytes (Figure 4A). IF confocal microscopy experiments indicated a reduced mGlur5 protein translation in SOD1^G93A^ astrocytes when exposed to the anti-mGluR5 ASO (Supplementary Figure S5).

Interestingly, GFAP and S100β expression was reduced in SOD1^G93A^ spinal cord astrocytes acutely treated with the ASO (Figure 4B–E) in comparison to the untreated or control ASO-treated SOD1^G93A^ cultured cells, indicating that also the acute genetic ablation of mGluR5 expression can modulate the reactive astrogliosis in SOD1^G93A^ astrocytes.

We also tested the pharmacological efficacy of the highly selective mGluR5 NAM CTEP, a compound optimized for in vivo treatments in rodents [67] and already tested in mouse models of several neurodegenerative diseases [86,87]. We exposed astrocytes from SOD1^G93A^ mice to 100 nM CTEP for 7 days. Confocal microscopy evidenced that the treatment with CTEP slightly, but significantly, reduced the GFAP (Figure 4F–H) and S100β (Figure 4G–I) expression in SOD1^G93A^ astrocytes, highlighting the positive effect of the pharmacological mGluR5 modulation in tuning the altered reactive phenotype of SOD1^G93A^ astrocytes.

### 3.5. Genetic Downregulation of mGluR5 Dampens NLRP-3 Inflammasome and Pro-Inflammatory Cytokines

After demonstrating the beneficial effects of the genetic mGluR5 downregulation on the altered activation state of spinal cord ALS astrocytes, we verified the impact on the neuroinflammatory astrocyte phenotype. NLRP-3 is an inflammasome component upregulated in ALS patients and mouse models of the disease [88,89]. In addition, dysregulation of cytokine production and secretion by astrocytes has been observed in ALS [90,91].

**Figure 5:**
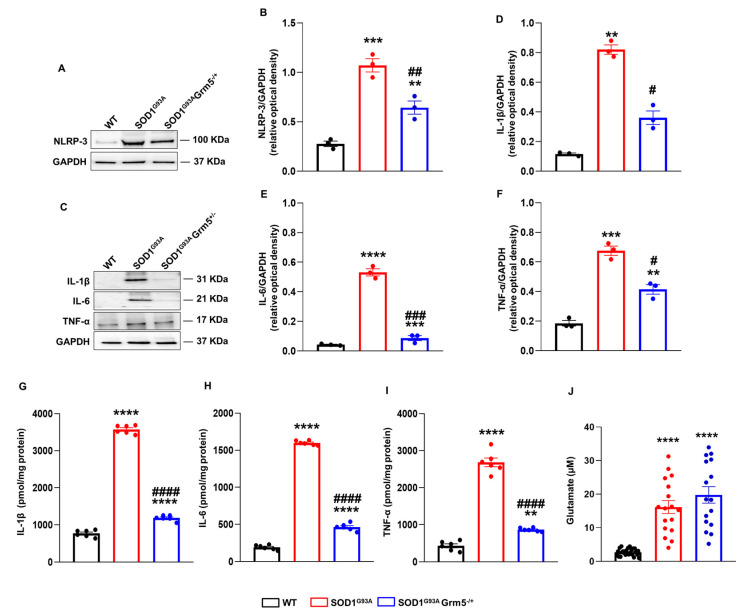
NLRP-3 inflammasome complex expression, cytokines and glutamate release in the culture medium of spinal cord astrocytes cultured from adult WT, SOD1^G93A^ and SOD1^G93A^Grm5^−/+^ mice. (**A**–**C**) Representative Western blot (WB) immunoreactive NLRP-3 (**A**) and IL-1β, IL-6, and TNF-α (**C**) bands. (**B**,**D**–**F**) Quantitative representation of WB densitometric signals of (**B**) NLRP-3, (**D**) IL-1β, (**E**) IL-6, and (**F**) TNF-α. Protein band density was normalized to GAPDH as a housekeeping protein. Data are means ± s.e.m of *n* = 3 independent experiments. ** *p* < 0.01, *** *p* < 0.001 and **** *p* < 0.0001 vs. WT astrocytes; # *p* < 0.05, ## *p* < 0.01 and ### *p* < 0.001 vs. SOD1^G93A^ astrocytes (F_(3,8)_ = 55.83, F_(2,6)_ = 23.18, F_(2,6)_ = 106.2 and F_(2,6)_ = 38.49 for NLRP-3, IL-1β, IL-6 and TNF-α respectively; one-way ANOVA followed by Tukey’s multi-comparison test). (**G**–**I**) Enzyme-linked immunosorbent (ELISA) assay of the (**G**) IL-1β, (**H**) IL-6, and (**I**) TNF-α released in the astrocyte culture medium. Cell culture medium was collected after 24 h, and the inflammatory cytokine content was measured with specific ELISA kits and expressed as pmol per mg of astrocyte’s total protein content in each well. Data means ± s.e.m of *n* = 6 independent experiments. ** *p* < 0.01 and **** *p* < 0.0001 vs. WT astrocytes; #### *p* < 0.0001 vs. SOD1^G93A^ astrocytes (F_(2,15)_ = 1563, F_(2,15)_ = 2426 and F_(2,15)_= 251.4, for IL-1β, IL-6 and TNF-α, respectively; one-way ANOVA followed by Tukey’s multi-comparison test). (**J**) Glu release in the astrocyte culture medium. Cell culture medium was replaced with Hepes-buffered physiological medium, which was collected after 4 h. Glu content was measured by HPLC after orthophthaldialdehyde derivatization and fluorometric detection. The physiological medium without cells contained 0.01 ± 0.002 µM of Glu (not shown). Glu is expressed as micromolar (µM) concentrations in each well, containing an average of 1 × 10^5^ cells. Data are means ± s.e.m of *n* = 16–27 wells (independent biological replicates). **** *p* < 0.0001 vs. WT astrocytes (F_(2,57)_ = 40.70; one-way ANOVA followed by Tukey’s multi-comparison test).

NLRP-3 expression was evaluated in WT, SOD1^G93A^ and SOD1^G93A^Grm5^−/+^ astrocytes by WB and confocal microscopy immunofluorescence semi-quantitative analyses. WB evidenced that spinal cord astrocytes cultured from late symptomatic SOD1^G93A^ mice undergo a 4-fold NLRP-increase with respect to WT astrocytes (Figure 5A,B). Interestingly, NLRP-3 expression in astrocytes cultured from SOD1^G93A^Grm5^−/+^ double mutant mice was significantly lower than that in SOD1^G93A^ astrocytes, although still more elevated than that in WT astrocytes (Figure 5A,B).

The expression and release of the pro-inflammatory cytokines IL-1β, IL-6, and TNF-α were analyzed by WB and ELISA, respectively. The IL-1β expression was dramatically increased in SOD1^G93A^ compared to WT astrocytes (Figure 5C,D). Conversely, SOD1^G93A^Grm5^−/+^ astrocytes showed a substantial reduction in IL-1β expression. IL-6 (Figure 5C–E) and TNF-α (Figure 5C–F) expression was also increased in SOD1^G93A^ compared to WT astrocytes, and the partial mGluR5 ablation, similar to IL-1β, significantly reduced their expression, even though these levels were still significantly higher compared to WT astrocytes. The cytokine concentrations were measured in the 24 h conditioned medium of WT, SOD1^G93A^ and SOD1^G93A^ Grm5^−/+^ cultured astrocytes by ELISA (Figure 5G–I). The IL-1β, IL-6, and TNF-α levels increased 4- to 7-fold in the conditioned medium of SOD1^G93A^ compared to WT astrocytes. In line with the reduced cellular cytokine expression, the over-release of IL-1β, IL-6 and TNF-α was almost abolished in spinal cord astrocytes cultured from adult SOD1^G93A^Grm5^−/+^ mice. However, the residual IL-1β, IL-6, and TNF-α releases from SOD1^G93A^Grm5^−/+^ astrocytes were still significantly higher compared to the WT conditions (Figure 5G–I).

Moreover, we measured Glu concentration in the conditioned medium of WT, SOD1^G93A^ and SOD1^G93A^ Grm5^−/+^ cultured astrocytes by HPLC. Glu was measured 4 h after substituting a saline physiological medium for the culture medium. Extracellular Glu content was significantly higher in the medium from SOD1^G93A^ and SOD1^G93A^ Grm5^−/+^ astrocytes compared to WT astrocyte supernatants. The partial mGluR5 ablation did not affect the excessive Glu release more than SOD1^G93A^ astrocytes (Figure 5J).

### 3.6. Genetic Downregulation of mGluR5 Positively Affects the Bioenergetic Metabolism in SOD1^G93A^ Spinal Cord Astrocytes

ALS is associated with altered cellular metabolism that involves MNs and other CNS cells, including astrocytes [92,93,94]. We investigated here whether the positive shift of the altered astrocyte phenotype obtained by the constitutive mGluR5 genetic downregulation was also paralleled by the amelioration of the energetic profile of these cells. We checked the oxygen consumption rate (OCR) and the ATP synthesis by the Fo-F1 ATP synthase in WT, SOD1^G93A^, and SOD1^G93A^Grm5^−/+^ astrocytes.

**Figure 6:**
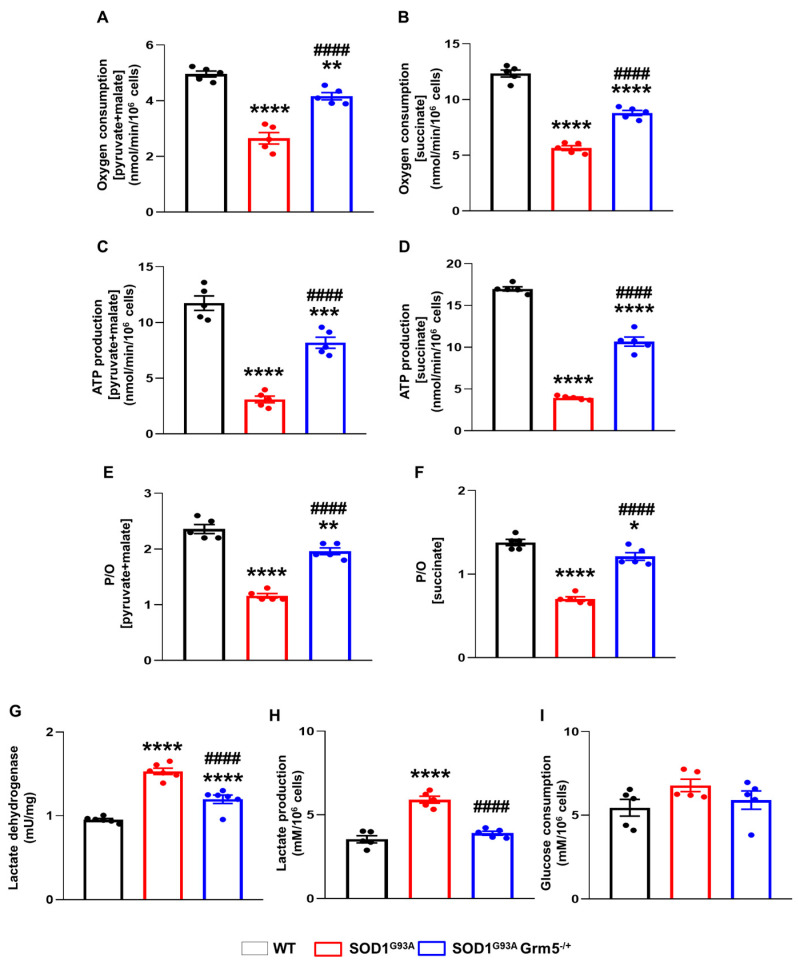
Energy metabolism in spinal cord astrocytes cultured from adult WT, SOD1^G93A^ and SOD1^G93A^Grm5^−/+^ mice. (**A**,**B**) Oxygen consumption. After permeabilization with 0.03% digitonin, WT, SOD1^G93A^, and SOD1^G93A^Grm5^−/+^ astrocytes were resuspended in a respiration medium and stimulated (**A**) with pyruvate (10 mM) + malate (5 mM) and ADP (0.1 mM) to evaluate the cellular respiration through the Complex I, III and IV pathway or (**B**) with succinate (20 mM) and ADP (0.1 mM) to investigate the activity of the Complex II, III, and IV pathway. The respiratory rate was expressed as nmol consumed oxygen/min/10^6^ cells. Data are means ± s.e.m of *n* = 5 independent experiments run in triplicate. ** *p* < 0.01 and **** *p* < 0.0001 vs. WT astrocytes; #### *p* < 0.0001 vs. SOD1^G93A^ astrocytes (pyruvate + malate: F_(2,12)_ = 59.22; succinate: F_(2,12)_ = 173.5; one-way ANOVA followed by Tukey’s multi-comparison test). (**C**,**D**) ATP synthesis by F_o_-F_1_ ATP synthase evaluated after stimulation (**C**) with pyruvate (10 mM) + malate (5 mM) or (**D**) with succinate (20 mM), and ADP (0.1 mM) in both conditions. Data are expressed as nmol ATP produced/min/10^6^ cells and are means ± s.e.m of *n* = 5 independent experiments. *** *p* < 0.001 and **** *p* < 0.0001 vs. WT astrocytes; #### *p* < 0.0001 vs. SOD1^G93A^ astrocytes (pyruvate and malate: F_(2,12)_ = 74.56; succinate: F_(2,12)_ = 344.6; one-way ANOVA followed by Tukey’s multi-comparison test). (**E**,**F**) P/O ratio as an OxPhos efficiency marker. The P/O was calculated as the ratio between the concentration of the produced ATP and the amount of consumed oxygen in the presence of (**E**) pyruvate (10 mM) + malate (5 mM) and ADP (0.1 mM) or (**F**) succinate (20 mM) and ADP (0.1 mM). Data are means ± s.e.m of *n* = 5 independent experiments run in triplicate. * *p* < 0.05, ** *p* < 0.01 and **** *p* < 0.0001 vs. WT astrocytes; #### *p* < 0.0001 vs. SOD1^G93A^ astrocytes (F_(2,12)_ = 94.92 for pyruvate and malate; succinate: F_(2,12)_ = 88.25; one-way ANOVA followed by Tukey’s multi-comparison test). (**G**) Lactate dehydrogenase (LDH) activity. LDH activity is expressed as international milliunits (mU/mg), corresponding to the nanomoles of substrate catalyzed in 1 min per mg of protein. Data are means ± s.e.m of *n* = 5 independent experiments, run in triplicate. **** *p* < 0.0001 vs. WT astrocytes; #### *p* < 0.0001 vs. SOD1^G93A^ astrocytes (F_(2,14)_ = 129; one-way ANOVA followed by Tukey’s multi-comparison test). (**H**) Lactate and (**I**) glucose concentrations were assayed spectrophotometrically in the culture medium. Data are expressed as mM concentration per 10^6^ cells and are means ± s.e.m of *n* = 5 independent experiments. **** *p* < 0.0001 vs. WT astrocytes; #### *p* < 0.0001 vs. SOD1^G93A^ astrocytes (F_(2,12)_ = 47.43; one-way ANOVA followed by Tukey’s multi-comparison test).

As expected, SOD1^G93A^ spinal cord adult astrocytes displayed a significant reduction in the OCR in the presence of pyruvate and malate or succinate compared to WT astrocytes. This reduction was partially reverted in SOD1^G93A^Grm5^−/+^ astrocytes after stimulation with both substrates (Figure 6A,B). A similar pattern was observed when monitoring the aerobic ATP synthesis. Indeed, SOD1^G93A^ astrocytes displayed a significant reduction in ATP production in the presence of pyruvate and malate (Figure 6C) or succinate (Figure 6D) compared to WT astrocytes. As observed in OCR experiments, the energetic alteration was partially reverted in SOD1^G93A^Grm5^−/+^ astrocytes. The oxidative phosphorylation efficiency, in terms of P/O values, was significantly reduced in SOD1^G93A^ astrocytes after stimulus with pyruvate and malate (Figure 6E) or succinate (Figure 6F), indicating a reduction in OxPhos efficiency with respect to WT astrocytes. Conversely, these values were significantly increased in SOD1^G93A^Grm5^−/+^ to an extent similar to that in WT astrocytes after the same respiratory substrates’ stimulus (Figure 6E,F), suggesting that SOD1^G93A^ astrocytes are characterized by the uncoupling between oxygen consumption and ATP synthesis, which is rescued by the partial reduction in mGluR5.

The enhanced anaerobic glycolytic flux, representing the prominent metabolism in astrocytes for ATP production [95,96] may compensate for reduced OxPhos-driven ATP synthesis, although less efficiently than aerobic metabolism. Data show that the activity of LDH, a key glycolytic enzyme (Figure 6G), and lactate production (Figure 6H) were augmented in SOD1^G93A^ astrocytes with respect to WT cells, indicating a partial compensatory effect in response to the OxPhos impairment. SOD1^G93A^Grm5^−/+^ astrocytes also displayed higher LDH activity (Figure 6G) than WT astrocytes, although their LDH activity was lower than that of SOD1^G93A^ astrocytes. Conversely, lactate production was normalized in SOD1^G93A^Grm5^−/+^ astrocytes (Figure 6H). In addition, glucose consumption was comparable in WT, SOD1^G93A^, and SOD1^G93A^Grm5^−/+^ astrocytes (Figure 6I).

### 3.7. The Genetic Downregulation of mGluR5 Reduces the Neurotoxicity of SOD1^G93A^ Astrocytes towards Spinal MNs

**Figure 7:**
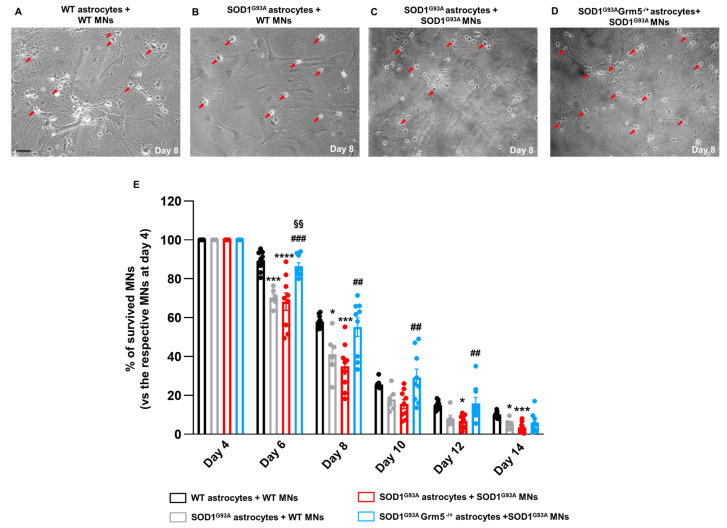
Survival of WT and SOD1^G93A^ MNs co-cultured with adult astrocytes from WT, SOD1^G93A^ and SOD1^G93A^Grm5^−/+^ mice. (**A**–**D**) Representative phase-contrast microscopy images (100×) of (**A**) WT MNs co-cultured with WT astrocytes, (**B**) WT MNs plated with SOD1^G93A^ astrocytes, (**C**) SOD1^G93A^ MNs plated with SOD1^G93A^ astrocytes and (**D**) SOD1^G93A^ MNs plated with SOD1^G93A^Grm5^−/+^ astrocytes; scale bar: 50 µm. (**E**) Quantification of MN viability. MNs were isolated from the spinal cord of WT and SOD1^G93A^ E13.5 mouse embryos and seeded on mature adult astrocyte cultures as described above. MNs were counted in a 1 cm^2^ area starting from day 4 after seeding, three times a week, for 14 days and expressed as percent (%) MN survival vs. the respective number of MNs counted on day 4. Data are means *±* SEM of *n* = 6–9 independent experiments. * *p* < 0.05, *** *p* < 0.001 and **** *p* < 0.0001 vs. WT MNs co-cultured with WT astrocytes; ## *p* < 0.01 and ### *p* < 0.001 vs. SOD1^G93A^ MNs co-cultured with SOD1^G93A^ astrocytes; §§ *p* < 0.01 vs. WT MNs co-cultured with SOD1^G93A^ astrocytes (day 6: F_(3,29)_ = 14.41; day 8: F_(3,29)_ = 8.820; day 10: F_(3,29)_ = 5.169; day 12: F_(3,29)_ = 6.032; day 14: F_(3,29)_ = 6.604; one-way ANOVA followed by Tukey’s multi-comparison test).

To determine whether the phenotypic amelioration obtained by the genetic downregulation of mGluR5 in SOD1^G93A^Grm5^−/+^ spinal cord astrocytes could reduce their neurotoxicity towards MNs, we set up mouse primary astrocyte/MN co-cultures. Therefore, we plated spinal cord astrocytes isolated from adult WT, SOD1^G93A^ or SOD1^G93A^Grm5^−/+^ mice. Then, spinal MNs isolated from WT or SOD1^G93A^ mouse embryos were seeded on astrocytes to obtain four different co-cultures: WT astrocytes/WT MNs, WT astrocytes/SOD1^G93A^ MNs, SOD1^G93A^ astrocytes/SOD1^G93A^ MNs, and SOD1^G93A^Grm5^−/+^ astrocytes/SOD1^G93A^ MNs (Figure 7A–D; representative images at day 8 of co-culture). MN viability was assessed between 4 and 14 days of co-culture and expressed as % of controls (total number of MNs at day 4). On day 4, the MN number was comparable in the different co-cultures. From day 6 onwards, we observed a constant decrease in viable MNs under all experimental conditions (Figure 7E), with almost 90% of neuronal loss at day 14. As expected, MN viability was significantly lower when co-cultured with SOD1^G93A^ astrocytes compared to the WT control co-cultures, at almost all the time points. Of note, the number of viable SOD1^G93A^ MNs co-cultured with SOD1^G93A^Grm5^−/+^ astrocytes was significantly higher and even superimposable to WT control conditions at each time point (Figure 7E).

## 4. Discussion

mGluR1 and mGluR5 are expressed at the synaptic level in neurons [97], other than in astrocytes, microglia and oligodendrocytes [78,98,99]. Glial cells regulate several altered cellular processes in ALS, thus playing a pivotal role in the complex scenario of the disease [46,48,49,100,101,102,103]. While astrocytes express low mGluR1 and mGluR5 levels under physiological conditions, reactive glial cells show higher receptor expression in the spinal cord of ALS patients [46]. mGluR5 overexpression was also detected in the striatum, hippocampus, frontal cortex and spinal cord of the SOD1^G93A^ mouse model of ALS, starting from the pre-symptomatic stages and during the disease progression [30,47,49]. mGluR5 is the most characterized Group I mGluRs, which likely correlates with the ALS pathology and actively modulates the glial response, affecting the local excitatory tone [76,104]. mGluR5 sustains neuronal growth, regulates synaptic activity and provides neuroprotection [105]. Its activation leads to several effects, such as astrocyte proliferation [106], the release of BDNF [107] and glio-transmitters such as ATP and Glu [108,109], modulation of inflammatory responses [110] and regulation of plasma membrane transporters and Glu uptake [45,111]. Thus, mGluR5 emerges as one leading actor that triggers the astroglial altered activation state and damage [48,49]. Elevated mGluR5 immunoreactivity is present in astrocytes derived from autoptic specimens of sALS patients and the spinal cord of SOD1^G93A^ mice [46]. Similar findings have also been reported in other models of neurological diseases, such as Huntington’s disease [112], Alzheimer’s disease [113], epilepsy [114] and fragile X syndrome [115], and in cultured astrocytes exposed to metabolic stress [116].

In our previous in vivo studies, both the knocking down [50] and knocking out [54] of mGluR5 ameliorated the pathological phenotype of SOD1^G93A^ mice and reduced astrocyte activation. To provide a translational value to these findings, we recently reported that the oral treatment of SOD1^G93A^ mice with the highly selective mGluR5 NAM CTEP effectively reproduced the beneficial outcomes of the genetic approach, slowing down the progression of the pathology, with a significant MN preservation and reduced astrocyte and microglia activation [51]. Due to the lack of knowledge of the effect of the mGluR5 genetic downregulation on specific CNS cell types, we here investigated primary astrocyte cell cultures prepared from the spinal cord of SOD1^G93A^Grm5^−/+^ mice at the late stage of the disease (120 days old). We compared their phenotype with astrocytes cultured from age-matched SOD1^G93A^, WT and Grm5^−/+^ mice and their neurotoxic potential towards spinal MNs.

Despite the obvious difficulties of setting and maintaining adult astrocyte cultures with respect to neonatal primary astrocyte ones, our choice of using these cells is grounded on the belief that the in vivo maturation during disease progression and the chronic exposure to a noxious environment during the different disease steps better recapitulate the cellular and molecular modifications which astrocytes undergo in situ. For this reason, we believe that adult mouse-derived spinal cord astrocytes represent a valuable in vitro experimental tool for studying symptomatic ALS mechanisms. Indeed, the in vitro activation state of our late symptomatic SOD1^G93A^ mouse-derived astrocytes can be attributed to the in vivo chronic exposure to the surrounding pathological milieu, which shapes their phenotype. Conversely, the amelioration registered in SOD1^G93A^Grm5^−/+^ astrocytes can be considered a direct consequence of the in vivo dampening of the contribution of mGluR5 to the cellular altered phenotype.

We previously proved that the mGluR5 expression was reduced in spinal cord homogenates from Grm5^−/+^ and SOD1^G93A^Grm5^−/+^ mice [50]. With the current experiments, we demonstrated that this reduction also takes place in spinal cord astrocytes. While SOD1^G93A^ astrocytes displayed high levels of mRNA encoding for mGluR5 and overexpressed the protein compared to WT astrocytes, SOD1^G93A^Grm5^−/+^ astrocytes showed a lower level of mRNA and total protein. Moreover, the constitutive, genetic downregulation effectively determined a reduced expression of mGluR5 at the plasma membrane of spinal cord astrocytes, significantly reducing the number of metabotropic receptors available for the endogenous ligand, thus dampening the excitatory glutamate transmission.

In physiological conditions, astrocyte activation represents a protective cell attempt in the case of acute damage. However, astrocytes gain an altered reactive phenotype in neurodegenerative diseases, such as ALS, that is toxic for the surrounding cells, particularly for neurons [117,118,119]. In line with the literature [82,120,121] and our previous results [56], we here demonstrated that spinal cord astrocytes from adult symptomatic SOD1^G93A^ mice overexpressed the cellular activation markers GFAP, vimentin and S100β, demonstrating that they preserve the activated phenotype in vitro, even after several DIV.

Remarkably, the genetic downregulation of mGluR5 in SOD1^G93A^Grm5^−/+^ astrocytes shifted the cell phenotype to a reduced activation state. No significant differences were detected when comparing WT and Grm5^−/+^ spinal cord astrocytes. We tried to replicate the constitutive in vivo effects by acutely reducing mGluR5 activity in in vitro experiments. ASO technology effectively treats neuromuscular diseases such as spinal muscular atrophy, Duchenne muscular dystrophy and ALS [12,122,123,124]. We showed here that the in vitro exposure of SOD1^G93A^ spinal cord astrocytes to antisense oligonucleotide anti-mGluR5 mimicked the in vivo mGluR5 genetic downregulation by reducing the expression of astroglial reactive markers. Pharmacologically reducing mGluR5 activation by the negative allosteric modulator CTEP attained the same results. Of note, this drug is representative of the therapeutic class of experimental mGluR5 NAMs recently under investigation in phase II/III clinical trials for neurological diseases other than ALS [125,126,127,128,129], with promising therapeutic and toxicological profiles [127,130,131]. The present and our previous findings could foster the potential repurposing of these drugs for ALS therapy.

We previously showed that [Ca^2+^]_i_ was significantly increased in synaptic nerve terminals purified from the spinal cord of SOD1^G93A^ mice, both at pre-symptomatic and symptomatic disease stages [15,29]. We also showed that the in vivo constitutive, genetic ablation of mGluR5 in SOD1^G93A^ mice significantly reduced [Ca^2+^]_i_ in spinal cord synaptosomes [50]. The normalization of [Ca^2+^]_i_ occurred in resting conditions and after depolarization by 15 mM KCl, or upon activation of group I metabotropic glutamate receptors with the selective agonist 3,5-DHPG [50]. Moreover, we demonstrated that the aberrant Ca^2+^ overload is brain region-specific and directly correlates to the activation of the calpain/calpastatin system, leading to proteolytic processes and cell death even at a very early non-symptomatic ALS stage [132,133]. Thus, our published data indeed suggest a key role of mGluR5 in shaping intracellular [Ca^2+^]_i_ fluxes. In the present study, spinal cord astrocytes cultured from SOD1^G93A^ mice displayed an elevated [Ca^2+^]_i,_ which was reduced in astrocytes cultured from SOD1^G93A^Grm5^−/+^ mice, both under resting and 3,5-DHPG-stimulated conditions. Although [Ca^2+^]_i_ reduction in SOD1^G93A^Grm5^−/+^ astrocytes did not return to WT levels, since many other mechanisms can intervene in the homeostasis of the ion, our results highlight that reduced receptor expression at the plasma membrane reduces the Glu-induced mGluR5 pathways affecting the intracellular calcium mobilization. This phenomenon may likely be related to the beneficial modulation of the reactive state of ALS astrocytes.

Along with microglia, astrocytes regulate the innate immune response in the CNS [90,134], releasing soluble factors, such as Glu, nitric oxide and pro-inflammatory cytokines [135]. Cytokine production and secretion dysregulation have been observed in ALS and several other neurodegenerative disorders [90,91,136]. We recently confirmed this pathological aspect by detecting an increase in pro-inflammatory cytokine expression and secretion in the conditioned medium of spinal cord astrocytes cultured from late symptomatic SOD1^G93A^ mice [56]. In the present study, we confirmed that the synthesis and release of IL-1β, IL-6, and TNF-α dramatically increased in SOD1^G93A^ astrocytes. Additionally, we observed that reducing the expression of mGluR5 limited the production and release of the three pro-inflammatory cytokines, suggesting that the genetic approach to lowering mGluR5 expression also led to the amelioration of the inflammatory traits of the milieu surrounding MNs.

Another potential trigger of astrocyte activation is Glu [137], which is actively released by astrocytes, particularly in neurodegenerative diseases, including ALS [138,139]. These data support the idea that the excessive Glu concentration in the surrounding milieu participates in the over-activation of glutamatergic receptors exposed by astrocytes, including mGluR5. Therefore, the partial constitutive ablation of mGluR5, although it did not directly affect the release of Glu from astrocytes, indeed significantly reduces the probability of Glu activating mGluR5, thus making Glu the primary factor accounting for the changes observed after partial suppression of mGluR5.

The energetic metabolism impairment is another pathological aspect in ALS preclinical models and patients [90]. We confirmed that spinal cord astrocytes cultured from symptomatic SOD1^G93A^ ALS mice are characterized by altered aerobic metabolism caused by the uncoupling between oxygen consumption and ATP production. This OxPhos alteration increased the anaerobic glycolysis flux, as demonstrated by the increased LDH activity and lactate release in SOD1^G93A^ astrocytes compared to WT cells. However, the basal astrocytes’ energy metabolism is already highly devoted to lactate production to sustain neurons [140,141]. Interestingly, paralleling the beneficial shift of the astrocyte reactive and inflammatory state, the metabolic dysfunctions were reverted in the SOD1^G93A^ Grm5^−/+^ astrocytes, supporting the idea that the mGluR5 genetic downregulation can play a pivotal role in positively modulating astrocyte energy metabolism dysregulation in ALS. Indeed, when OxPhos is strongly uncoupled, as observed in SOD1^G93A^ astrocytes, the system dramatically increases electron leakage through the respiratory complexes favoring the production of reactive oxygen species [72,142]. Although OCR and ATP synthesis were only partially rescued in SOD1^G93A^ Grm5^−/+^ astrocytes, the complete recovery of OxPhos efficiency suggests a reduction in oxidative stress production and a possible contribution to dampening the detrimental environmental impact that directly participates in MN degeneration and death.

Lastly, we determined whether the reduction in mGluR5 expression in SOD1^G93A^ mouse-derived spinal cord astrocytes and the overall amelioration of their phenotype impact spinal MN viability. We set up mouse primary MN co-cultures, seeding WT or SOD1^G93A^ mouse embryonic spinal cord MNs on astrocytes prepared from the spinal cord of SOD1^G93A^ or SOD1^G93A^Grm5^−/+^ mice, and assessed the MN viability for 10 days starting at co-culture day 4. MN viability declined faster when cultured with SOD1^G93A^ than WT astrocytes. Interestingly, as for SOD1^G93A^ MNs, WT MN viability was also reduced when exposed to SOD1^G93A^ astrocytes. These data indicate that WT and SOD1^G93A^ MNs were similarly affected by SOD1^G93A^ astrocyte toxicity, confirming that MN death may rely on the detrimental impact of SOD1 ^G93A^ expression in astrocytes. Instead, the rate of SOD1^G93A^ MN death was significantly reduced when co-cultured with SOD1^G93A^Grm5^−/+^ astrocytes, demonstrating a causal and beneficial connection between dampening the astrocytes’ reactive phenotype through mGluR5 negative modulation and MN survival improvement.

## 5. Conclusions

In conclusion, our results provide, for the first time, compelling evidence of the positive impact of mGluR5 genetic ablation on the astrocytes’ cellular phenotype in the SOD1^G93A^ mouse model of ALS. Indeed, spinal cord astrocytes cultured from late symptomatic SOD1^G93A^ mice show an altered reactive state that can be effectively modulated by selectively reducing the expression of mGluR5. Most attractively, the genetic mGluR5 downregulation, restoring the astrocyte phenotype, translates into reduced toxicity towards MNs.

Considering the encouraging results that we have reported in different preclinical in vivo studies [50,51,54], and the data here presented, we propose mGluR5 as a promising target for pharmacological interventions in ALS. The present data show a relevant astrocyte involvement in the positive impact obtained by the mGluR5 genetic downregulation in ALS mice, thus strongly supporting the hypothesis that selective approaches aimed at targeting a specific cell population may represent an intriguing therapeutic strategy. Here we reported that the selective dampening of mGluR5 in astrocytes counteracted the altered reactive phenotype of these cells and preserved MN loss during ALS progression. Finally, we believe that the present evidence should also be considered for other neurological and neurodegenerative diseases that share features with ALS or where astrocytes play a crucial role in sustaining the disease processes.

## Data Availability

The raw data and datasets are available from the corresponding author upon reasonable request.

## Supplementary Materials

The following supporting information can be downloaded at: https://www.mdpi.com/article/10.3390/cells12151952/s1, Supplementary Figure S1: Schematic representation of the protocol used to generate spinal cord primary astrocyte cell culture isolated from WT, Grm5^−/+^, SOD1^G93A^ and SOD1^G93A^Grm5^−/+^ adult mice and astrocyte cell culture purity. Supplementary Figure S2: Confocal microscopy representative images showing MN/astrocyte co-cultures labeled with specific MN markers. Supplementary Figure S3: RT-PCR quantification of mGluR5 mRNA in WT, Grm5^−/+^, SOD1^G93A^ and SOD1^G93A^Grm5^−/+^ astrocytes. Supplementary Figure S4: Expression and cellular localization of misfolded hSOD1 in spinal cord astrocytes cultured from adult WT, Grm5^−/+^, SOD1^G93A^ and SOD1^G93A^Grm5^−/+^ mice. Supplementary Figure S5: Expression of mGluR5 in spinal cord SOD1^G93A^ astrocytes exposed to antisense oligonucleotide anti-mGluR5. Supplementary Table S1: List of primary and secondary antibodies used for WB experiments. Supplementary Table S2: List of primary and secondary antibodies used for immunofluorescence analyses. Supplementary Table S3. Fluorescence intensity of the signals for GAPDH immunostaining calculated as corrected total cell fluorescence (CTCF—arbitrary units) and expressed as integrated density.
