# Supplementary material for: Genetic Downregulation of the Metabotropic Glutamate Receptor Type 5 Dampens the Reactive and Neurotoxic Phenotype of Adult ALS Astrocytes
Source: Cells. 2023 Jul 27;12(15):1952. doi: 10.3390/cells12151952 (PMC10416852; doi:10.3390/cells12151952)
Supplement: Supplementary file 1 [file cells-12-01952-s001.zip › cells-2457247-supplementary.pdf]

Supplementary materials

Genetic Downregulation of the Metabotropic Glutamate Receptor Type 5 Dampens the Reactive and Neurotoxic Phenotype of Adult ALS Astrocytes

Carola Torazza [1], Francesca Provenzano [1], Elena Gallia [1], Maria Cerminara [2,3], Matilde Balbi [1], Tiziana Bonifacino [1,4], Sara Tessitore [1], Silvia Ravera [5], Cesare Usai [6], Ilaria Musante [3], Aldamaria Puliti [2,3], Ludo Van Den Bosch [7,8], Paymaan Jafar-nejad[9], Frank Rigo[9], Marco Milanese [1,10] *, and Giambattista Bonanno [1]

Supplementary materials

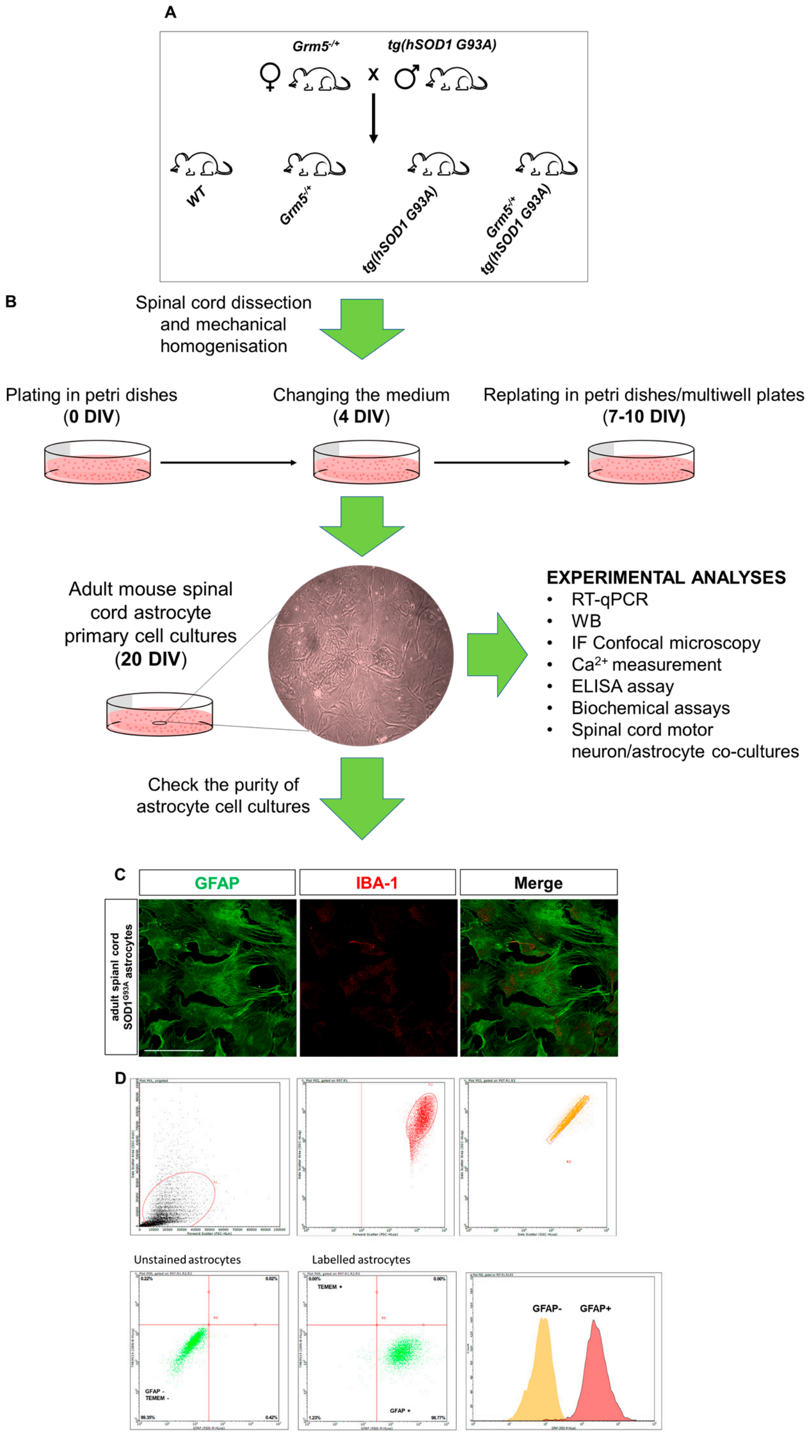

Supplementary Figure S1. Schematic representation of spinal cord astrocyte primary cell culture preparation, purity check and performed experimental analyses. (**A**) The four mouse littermates (WT, Grm5$^{-/+}$, SOD1^{G93A}, and SOD1^{G93A}Grm5$^{-/+}$ double mutant mice) were obtained by crossing Grm5$^{-/+}$ and SOD1^{G93A} mice as previously reported [1]. (**B**) Schematic representation of spinal astrocyte primary cell cultures preparation obtained by the four littermates described above, representative phase contrast microscopy image of mature astrocyte cultures, and proposed experimental design. (**C**) Representative confocal microscopy images of astrocyte primary cell cultures from SOD1^{G93A} spinal cord adult mice stained with selective antibodies for GFAP (specific astrocyte marker; green fluorescence) and IBA1 (specific microglia marker; red fluorescence). Scale bar: 100 µm. (**D**) Representative flow cytometry dot plots and histogram chart relative to astrocyte primary cell cultures from SOD1^{G93A} spinal cord adult mice unstained or labelled with fluorophore-conjugated selective antibody for GFAP (red fluorescence) and TMEM119 (green fluorescence).

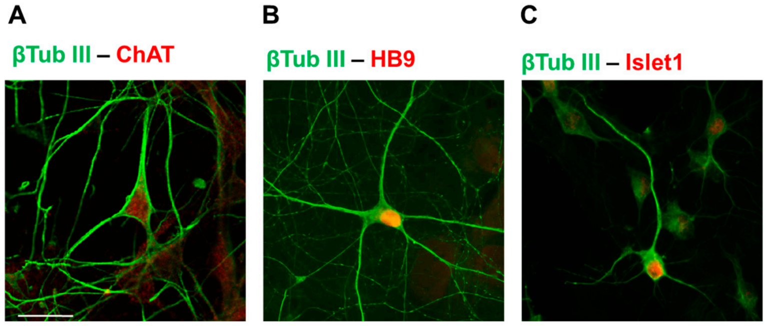

Supplementary Figure S2. Representative confocal microscopy images of spinal cord MN primary cell cultures labelled with specific markers. MNs were labelled with antibodies selective for beta-tubulin-III (βTub-III, green fluorescence) and the respective MN specific markers (red fluorescence) (**A**) choline acetyltransferase (ChAT,) (**B**), homeobox protein HB9 (HB9), and (**C**) homeobox protein Islet1 (Islet1). Scale bar: 50 μm.

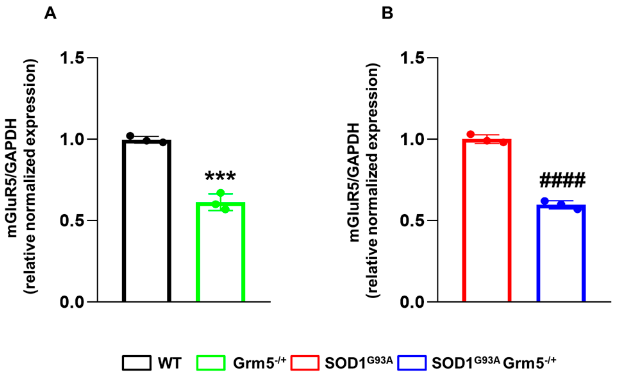

Supplementary Figure S3. RT-qPCR quantification of the mGluR5 mRNA in WT, Grm5$^{-/+}$, SOD1^{G93A} and SOD1^{G93A}Grm5$^{-/+}$ astrocytes. **(A)** WT and Grm5$^{-/+}$ astrocytes; (**B**) SOD1^{G93A} and SOD1^{G93A}Grm5$^{-/+}$ astrocytes. Data are means ± s.e.m of n=3 independent experiments run in triplicate. The mRNA expression of *Grm5* in WT and SOD1^{G93A} astrocytes is reported as a unit. ***p<0.001 vs WT astrocytes; $^{####}$p<0.0001 vs SOD1^{G93A} astrocytes ($t_{(4)}$=11.99; two-tailed Student's t-test).

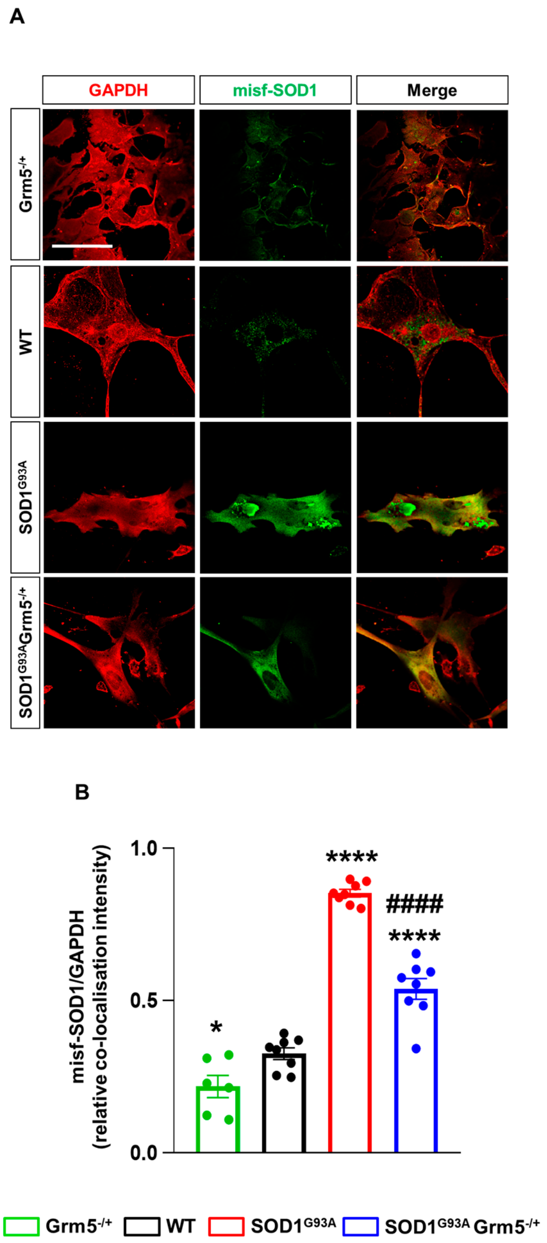

Supplementary Figure S4. Expression and cellular localization of misfolded hSOD1 in spinal cord astrocytes cultured from adult WT, Grm5$^{-/+}$, SOD1^{G93A}, and SOD1^{G93A}Grm5$^{-/+}$ mice. (**A**) Representative confocal microscopy immunocytochemical images of misfolded human SOD1 (hSOD1, green fluorescence) and GAPDH (red fluorescence). Grm5$^{-/+}$, WT, SOD1^{G93A}, and SOD1^{G93A}Grm5$^{-/+}$ spinal cord astrocytes were fixed, permeabilized and incubated with appropriate primary and fluorescent secondary antibodies. Images were acquired by confocal microscopy. Scale bar: 100 μm. (**B**) The quantitative representation of hSOD1 expression, calculated as the relative fluorescence intensity of the protein of interest, co-localized with the reference protein GAPDH. Data are means ± s.e.m of n=6-8 independent experiments. *$p<0.05$ and ****$p<0.0001$ vs WT astrocytes; $^{####}p<0.0001$ vs SOD1^{G93A} astrocytes; ($F_{(3,26)}$=115.5; one way ANOVA followed by Tukey's multi comparison test).

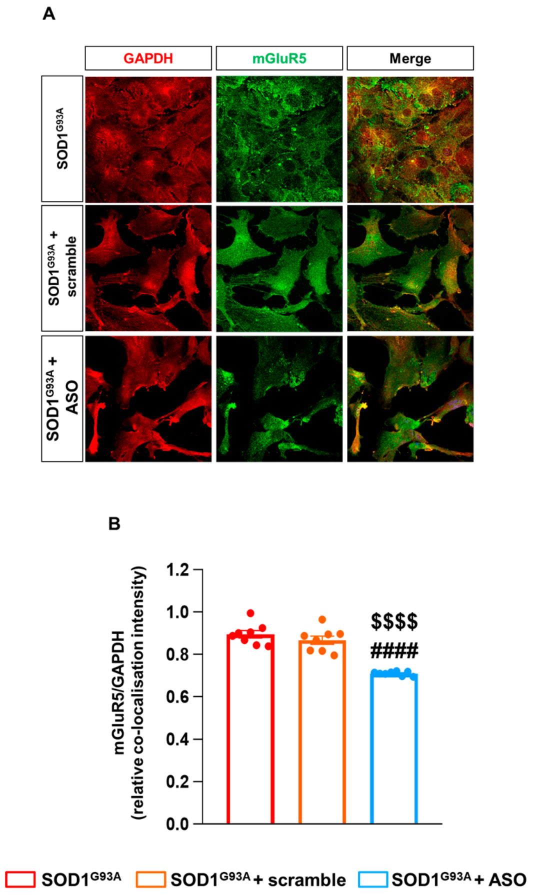

Supplementary Figure S5. Expression of mGluR5 in spinal cord SOD1^{G93A} astrocytes exposed to antisense oligonucleotide anti-mGluR5. (**A**) Representative confocal microscopy immunocytochemical images of mGluR5 (green fluorescence) and GAPDH (red fluorescence) in untreated, scramble- and ASO-treated SOD1^{G93A} astrocytes. Scale bar: 100 µm. Astrocytes were labelled with appropriate primary and fluorescent secondary antibodies, and the images were acquired by confocal microscopy. (**B**) Quantitative representation of mGluR5 total protein expression, calculated as the relative fluorescence intensity of the protein of interest co-localized with the reference protein GAPDH. Data are means ± s.e.m of n=8 independent experiments. $^{####}p<0.0001$ vs untreated SOD1^{G93A} astrocytes and $^{\$\$\$\$}p<0.0001$ vs scramble-treated SOD1^{G93A} astrocytes ($F_{(2,21)}=42.52$; one-way ANOVA followed by Tukey's multi-comparison test).

Supplementary Table S1. List of primary and secondary antibodies used for western blot experiments.

PRIMARY ANTIBODY	WORKING DILUTION	PRODUCER AND CATALOGUE NUMBER
mouse monoclonal anti-GFAP antibody	1:1000	Sigma Aldrich, Cat# G3893
mouse monoclonal anti-S100β antibody	1:100	Merck Millipore, Cat# MAB079
mouse monoclonal anti-Vimentin antibody	1:1000	Sigma Aldrich, Cat# V2258
rabbit recombinant monoclonal anti-NLRP-3 antibody	1:500	Abcam, Cat# ab210491
goat polyclonal anti-IL-1β antibody	1:500	Sigma Aldrich, Cat# I3767
goat polyclonal anti-TNF-α antibody	1:1000	Sigma Aldrich, Cat# T0938
chicken polyclonal anti-IL-6 antibody	1:500	Sigma Aldrich, Cat# GW22495
mouse monoclonal anti-GAPDH antibody	1:10000	Sigma Aldrich, Cat# G8795
rabbit polyclonal anti-GAPDH antibody	1:10000	Sigma Aldrich, Cat# G9545
rabbit polyclonal anti-mGluR5 antibody	1:500	Abcam, Cat# ab53090
**SECONDARY ANTIBODY**	**WORKING DILUTION**	**PRODUCER AND CATALOGUE NUMBER**
goat polyclonal anti-mouse IgG (HRP) antibody	1:10000	Bio-Rad, Cat# 0300-0108P
goat polyclonal anti-rabbit IgG (HRP) antibody		Bio-Rad, Cat# STAR124P
rabbit polyclonal anti-goat IgG (HRP) antibody		Bio-Rad, Cat# 5160-2104
goat polyclonal anti-chicken IgG (HRP) antibody		Bio-Rad, Cat# AAI29

Supplementary Table S2. List of primary and secondary antibodies used for immunofluorescence analyses.

PRIMARY ANTIBODY	WORKING DILUTION	PRODUCER AND CATALOGUE NUMBER
rabbit polyclonal anti-mGluR5 (extracellular)-ATTO-594 antibody	1:500	Alomone labs, Cat# AGC-007-AR
Rabbit anti-Lectin, *Triticum vulgaris* antibody	1:1000	Sigma Aldrich, Cat# I3767
mouse monoclonal anti-GFAP antibody	1:1000	Sigma Aldrich, Cat# G3893
mouse monoclonal anti-S100β antibody	1:500	Merck Millipore, Cat# MAB079
Mouse monoclonal anti-Vimentin antibody	1:1000	Sigma Aldrich, Cat# V2258
mouse monoclonal anti-GAPDH antibody	1:1000	Sigma Aldrich, Cat# G8795
rabbit polyclonal anti-GAPDH antibody	1:1000	Sigma Aldrich, Cat# G9545
Rabbit anti-Iba1 antibody	1:500	FUJIFILM Wako Chemical Corporation, Cat# 016-20001
mouse monoclonal anti-misfolded human superoxide dismutase 1 (hSOD1) antibody	1:100	Medimabs, Cat# B8H10
**SECONDARY ANTIBODY**	**WORKING DILUTION**	**PRODUCER AND CATALOGUE NUMBER**
donkey anti-rabbit Alexa Fluor A488-conjugated	1:3000	Thermo fisher Scientific, Cat# R37118
donkey anti-mouse Alexa Fluor A488-conjugated		Thermo fisher Scientific, Cat# A21202
donkey anti-mouse Alexa Fluor A647-conjugated		Thermo fisher Scientific, Cat# A31571
donkey anti-rabbit Alexa Fluor A647-conjugated		Thermo fisher Scientific, Cat# A31573

Supplementary Table S3. Fluorescence intensity of the signals for GAPDH immunostaining calculated as corrected total cell fluorescence (CTCF - arbitrary units) and expressed as integrated density.

GAPDH CTCF (Corrected total cell fluorescence)				
	Grm5 +/-	*WT*	*SOD1G93A*	*SOD1G93A_Grm5+/-*
Figure 3E	*72.44* ± 1.10	*71.21* ± 1.21	*74.11* ± 2.06	*71.87* ± 1.76
Figure 3F	*83.25* ± 2.26	*83.79* ± 2.67	*83.90* ± 2.05	*83.30* ± 1.44
Figure 3G	*84.92* ± 2.02	*84.91* ± 2.04	*85.14* ± 2.40	*84.74* ± 1.67
	SOD1G93A	*SOD1G93A + scramble*	*SOD1G93A + ASO*	
Figure 4B	*76.47* ± 1.84	*76.63* ± 1.97	*76.24* ± 1.53	
Figure 4C	*83.34* ± 1.52	*82.70* ± 1.59	*82.79* ± 2.32	
	SOD1G93A +dmso	*SOD1G93A + CTEP*		
Figure 4F	*74.40* ± 2.15	*74.19* ± 2.07		
Figure 4G	*97.70* ± 1.74	*99.33* ± 3.16		
	Grm5 +/-	*WT*	*SOD1G93A*	*SOD1G93A_Grm5+/-*
Figure S4	*63.89* ± 3.80	*60.92* ± 4.19	*62.34* ± 2.77	*63.20* ± 2.96
	SOD1G93A	*SOD1G93A + scramble*	*SOD1G93A + ASO*	
Figure S5	*72.76* ± 2.40	*72.42* ± 2.20	*71.81* ± 3.08	
